# Ultrasound and ROS-responsive nanodroplets inhibit TCA cycle in hepatocellular carcinoma

**DOI:** 10.1186/s12951-026-04190-y

**Published:** 2026-03-01

**Authors:** Ting Zhao, Lu Guo, Ning Cong, Yading Zhao, Xiaoxuan Wang, Xinyu Zeng, Suyun Li, Rui Liu, Shuting Huang, Yuye Fu, Jie Li

**Affiliations:** 1 Department of Ultrasound, Qilu Hospital of Shandong University, Jinan, 250012 Shandong China; 2 Department of Ultrasound, Qilu Hospital (Qingdao) of Shandong University, Qingdao, 266035 Shandong China

**Keywords:** Nanodroplets, ROS-responsive, Ultrasound targeted microbubble destruction, Hepatocellular carcinoma, Tricarboxylic acid cycle

## Abstract

Abnormal energy metabolism patterns are common characteristics of various tumor cells, including hepatocellular carcinoma cells (HCC). HCC undergo metabolic reprogramming, upregulating the tricarboxylic acid (TCA) cycle and mitochondrial respiration to provide energy. Based on this, we constructed a novel reactive oxygen species (ROS)- and ultrasound-responsive nanodroplet (named sGTND), which were loaded with glucose-6-phosphate dehydrogenase (G6PD) siRNA and modified with glycyrrhetinic acid (GA) via GTC polymer. These sGTNDs have a particle size of approximately 300 nm, exhibit excellent biocompatibility and contrast-enhanced ultrasound imaging. By recognizing GA receptors, sGTNDs can be specifically targeted and enriched within HCC. Under the combined effect of ultrasound-targeted microbubble destruction (UTMD), sGTNDs release GA to activate the nuclear factor erythroid 2-related factor 2 (NRF2) while simultaneously releasing siRNA to inhibit G6PD. This dual action synergistically inhibits the TCA cycle, resulting in an antitumor efficacy of up to 95.1%, which is significantly higher than that observed in the control groups. Thus, the combination of sGTND and UTMD can effectively inhibit the TCA cycle through NRF2 activation and G6PD inhibition, which represents a promising therapeutic approach for HCC.

**Graphical abstract:**

**Supplementary Information:**

The online version contains supplementary material available at 10.1186/s12951-026-04190-y.

## Highlights

ROS- and US-responsive targeted sGTND was constructed.

sGTND possesses excellent biocompatibility and exhibits ROS-responsive drug release.

sGTND responds to US to achieve UTMD effects and CEUI imaging.

sGTND/UTMD combination inhibits TCA cycle through NRF2 activation and G6PD inhibition.

**Supplementary Information:**

The online version contains supplementary material available at 10.1186/s12951-026-04190-y.

## Graphical abstract

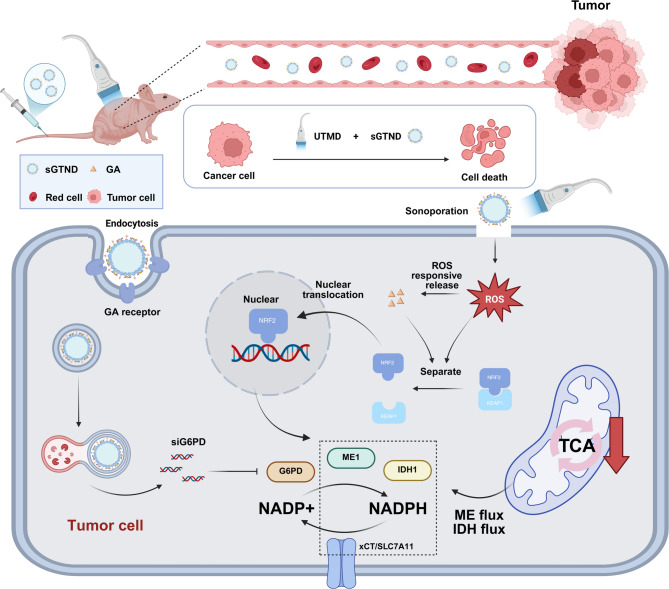

## Introduction

Hepatocellular carcinoma (HCC), is one of the most common and aggressive malignancies worldwide. It often develops silently in the early stages, with symptoms only appearing when the disease is advanced [1, 2]. Traditional treatments like surgery, chemotherapy, and radiation therapy have limitations in efficacy and can cause severe side effects. This highlights the urgent need to explore novel therapeutic strategies. In recent years, nanotechnology has shown great potential in cancer treatment, and the development of new nano-based therapies for HCC is of vital importance.

It was previously believed that tumor cells typically undergo metabolic reprogramming, utilizing aerobic glycolysis to generate energy while bypassing the tricarboxylic acid (TCA) cycle, which is known as the Warburg effect [3]. Warburg initially hypothesized that tumor cells exhibit mitochondrial defects that hinder aerobic respiration, leading to an increased reliance on glycolysis for energy production [4]. However, emerging evidence challenges this conception, suggesting that not all tumors rely predominantly on aerobic glycolysis. In fact, further studies indicate that mitochondrial function remains intact in cancer, with some tumor cells depending heavily on the TCA cycle for energy production and macromolecule synthesis [5–7]. For example, Guo et al. demonstrated that an increased influx of acetyl-coenzyme A into the TCA cycle results in enhanced energy production, which in turn supports the rapid proliferation of HCC cells and promotes tumor growth [8]. Therefore, inhibiting the TCA cycle is an effective strategy against tumors.

Nuclear Factor Erythroid 2-related Factor 2 (NRF2) is a transcription factor that regulates cellular defense against toxicity and oxidative damage by modulating the expression of genes involved in the oxidative stress response and drug detoxification [9]. Studies have demonstrated that tumors with NRF2 pathway activation rely on glucose-6-phosphate dehydrogenase (G6PD) to maintain TCA cycle homeostasis [10]. This dependence arises because nicotinamide adenine dinucleotide phosphate (NADPH) in cells is generated through three pathways: malic enzyme 1 (ME1), isocitrate dehydrogenase 1 (IDH1) and the pentose phosphate pathway (PPP), with the PPP being the primary source of NADPH [11]. Upon NRF2 activation, the expression of xCT/solute carrier family 7 member 11 (xCT/SLC7A11) increases, leading to substantial consumption of intracellular NADPH. To counteract oxidative stress, these cells depend on G6PD to produce NADPH. When G6PD is absent or insufficient, tumor cells utilize cytoplasmic ME1 and IDH1 to sustain redox homeostasis [12, 13]. As a result, TCA cycle metabolites such as malate and isocitrate are extensively effluxed from the mitochondria and consumed, thereby inhibiting the TCA cycle.

Glycyrrhetinic acid (GA), a pentacyclic triterpenoid saponin of the oleanane type, exhibits a range of significant pharmacological properties, including anti-inflammatory, anti-diabetic, anti-tumor, and anti-fibrotic effects [14]. Research has demonstrated that GA can induce the dissociation of NRF2 from Kelch-like ECH-associated protein 1 (KEAP1) and promote its nuclear translocation, thereby modulating the expression of downstream target genes [15]. Thus, GA can serve as an activator of the NRF2 pathway. Moreover, given that HCC cells express numerous GA receptors on their surface [16], GA can also function as a targeting molecule for liver cancer.

Based on the above, we hypothesize that activating the NRF2 signaling pathway with GA combined with siRNA-mediated suppression of G6PD expression could serve as an effective strategy to synergistically inhibit the TCA cycle, thereby offering a promising approach for treating tumors. Furthermore, how to effectively deliver GA and siRNA to tumor sites remains a key challenge. Traditional methods often suffer from low bioavailability, inadequate targeting, and undesirable side effects. Consequently, researchers are increasingly focused on discovering effective drug delivery systems (DDS) to improve drug efficacy and reducing toxicity. Among these, stimulus-responsive DDS has gained prominence due to its ability to precisely target therapeutic sites [17–19]. Ultrasound (US), as an external stimulus, offers several advantages, including noninvasiveness, spatiotemporal control, cavitation, and sonoporation effects [20]. US-responsive DDS refers to nanodroplets (NDs) with perfluorocarbon gas or liquid core. Under US irradiation, these NDs not only enhance US imaging for tumor visualization and therapeutic monitoring, but also generate ultrasound-targeted microbubble destruction (UTMD) effects, which promote drug release and uptake, and trigger various tumor biological responses, such as an upregulation of tumor reactive oxygen species (ROS) levels [21–23]. In addition to external stimuli, internal stimuli are also a crucial component of stimulus-responsive DDS. For example, ROS-responsive DDS have garnered significant attention in cancer research due to their potential for precise spatiotemporal drug release tailored to the high ROS levels within tumors, despite challenges such as heterogeneity in ROS distribution and endogenous supply limitations [24].

**Fig. 1:**
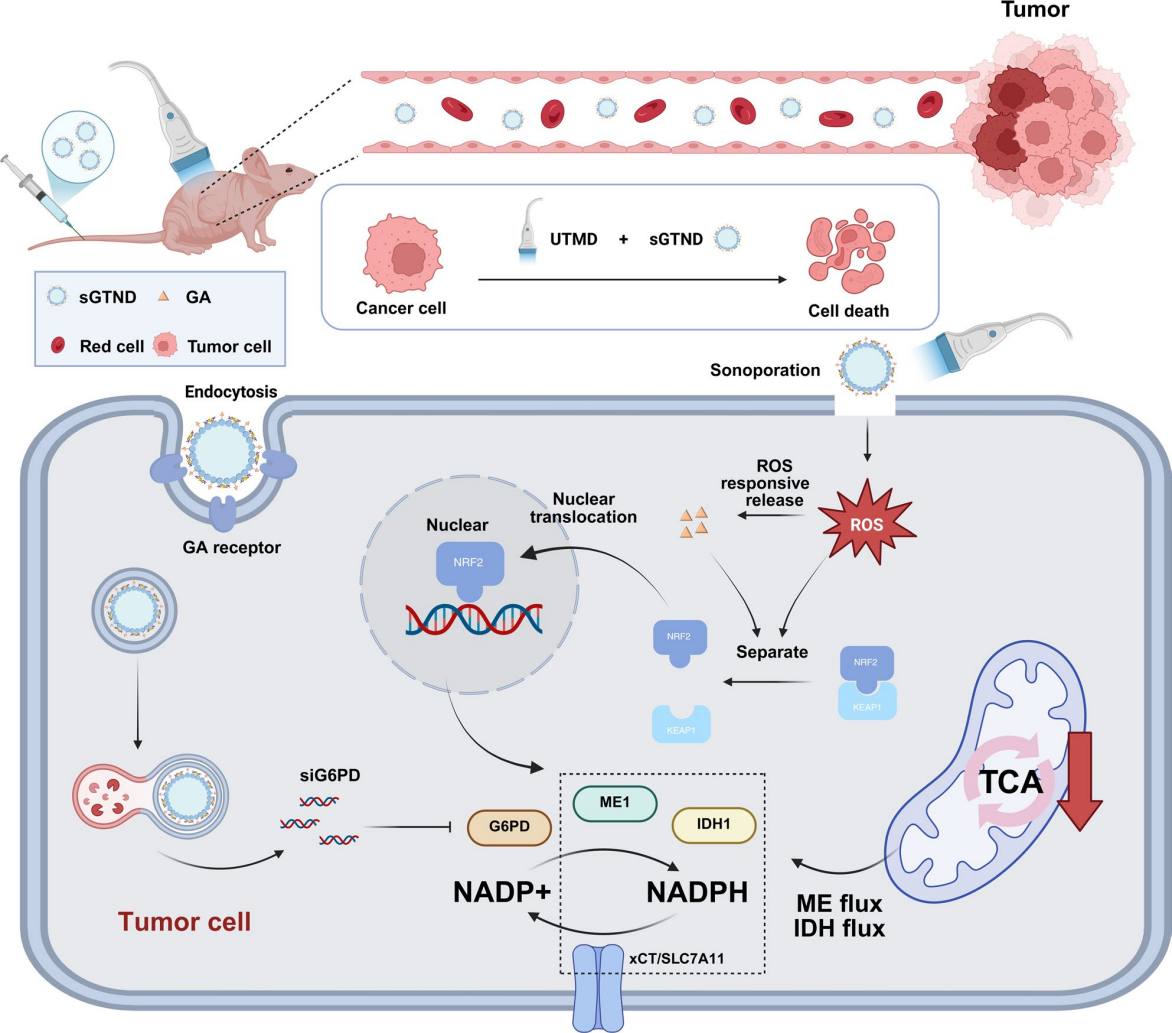
Schematic diagram of the antitumor effect of sGTND in combination with UTMD through inhibiting TCA cycle. sGTND specifically targets HCC cells via GA modification, and in combination with the sonoporation effect induced by UTMD, achieves efficient tumor targeting and cellular uptake. UTMD elevates intracellular ROS levels, thereby triggering the ROS-responsive release of GA and subsequent activation of the NRF2 signaling pathway. Activation of NRF2 leads to the upregulation of xCT/SLC7A11, which increases NADPH consumption. As a consequence, tumor cells become highly dependent on the PPP to maintain redox homeostasis. Meanwhile, together with the downregulation of G6PD, the key rate-limiting enzyme of the PPP, limits NADPH regeneration. This metabolic imbalance results in the depletion of key TCA cycle-associated metabolites, including ME and IDH, thereby suppressing TCA cycle activity and ultimately inducing tumor cell death

In this study, we designed and synthesized the novel GTC polymer that links GA and carboxymethyl chitosan (CMC) via a ROS-responsive thioacetal (TK) bond. Using this GTC polymer as the shell and perfluorohexane (PFH) as the core, polyethylenimine-G6PD siRNA (PEI-siG6PD) was encapsulated, thereby constructing the ND (named sGTND) via an emulsification/homogenization method. sGTND can specifically recognize HCC through GA modification, and in conjunction with the sonoporation effect of UTMD, achieve effective tumor targeting and cellular uptake. UTMD increases intracellular ROS levels and triggers the ROS-responsive release of GA, which in turn activates the NRF2 pathway. The effective activation of NRF2 and the subsequent reduction in G6PD expression deplete TCA cycle metabolites, thereby inhibiting the TCA cycle and ultimately inducing tumor cell death (Fig. 1). What’s more, as an US contrast agent, sGTND can achieve contrast-enhanced US imaging (CEUI), enabling specific tumor diagnosis. This combined strategy of sGTND with UTMD significantly inhibited TCA through NRF2 activation and G6PD inhibition, introducing a novel approach to the treatment of HCC.

## Materials and methods

### Material

1-ethyl-(3-dimethylaminopropyl) carbodiimide hydrochloride (EDC·HCl), N-hydroxysuccinimide (NHS), dimethyl sulfoxide (DMSO), GA and DCFH-DA were purchased from MedChemExpress (USA). NH_2_-TK-NH_2_ was obtained from Yusi Medical Technology (Chongqing, China). CMC (Degree of substitution ≥ 90%; MW = 318 kDa) was purchased from Macklin (Shanghai, China). Lecithin and PFH were obtained from Aladdin (Shanghai, China). Tween20 was purchased from Solar Bio (Beijing, China). Branched polyethylenimine (25KDa) was obtained from Sigma-Aldrich (USA). siG6PD and Cy5-siG6PD were purchased from GenePharma (Shanghai, China). FITC was obtained from SparkJade (Shandong, China). Rabbit anti-G6PD antibody, rabbit anti-β-actin antibody, rabbit anti-xCT/SLC7A11 antibody, and rabbit anti-NRF2 antibody were purchased from HuaBio (Hangzhou, China). Rabbit anti-ME1 antibody and rabbit anti-IDH1 antibody were obtained from Abclonal (Wuhan, China). NADP+/NADPH, malate, isocitrate, and fumarate content detection kits, as well as Edu594 cell proliferation kit, were purchased from Beyotime (Shanghai, China). AnnexinV- FITC/PI cell apoptosis kit was obtained from Vazyme (Nanjing, China).

### Synthesis and characterization of GTC polymer

To synthesize the GTC polymer, 11.8 mg of GA, 5.8 mg of EDC·HCl, and 3.5 mg of NHS were first dissolved in DMSO. The mixture was stirred at room temperature for 0.5 h, after which 7.3 mg of NH_2_-TK-NH_2_ was added. The reaction was continued by stirring at room temperature for an additional 24 h to obtain the GA-TK intermediate solution. Subsequently, 20.4 mg of CMC, 8.6 mg of EDC·HCl, and 5.2 mg of NHS were dissolved in deionized water. After stirring at room temperature for 0.5 h, this solution was combined with the GA-TK solution and stirred at room temperature for another 24 h to form the GTC product. The resulting product was transferred to a dialysis bag (MW 3500 Da) and dialyzed against deionized water for 24 h to remove unreacted starting materials and by-products. The water was changed every 4 h during the dialysis process. After dialysis, the product was centrifuged, and the precipitate was collected and lyophilized to obtain the final GTC product, which was stored at -20℃.

The coupling efficiency of GA was assessed by measuring the GA content in the supernatant at 257 nm using ultraviolet spectrophotometry (Shimadzu, Tokyo, Japan). The GA coupling rate was calculated using the following formula: GA coupling rate (%) = (Total amount of GA added - Amount of GA in the supernatant) / Total amount of GA added×100%.

The chemical structure and composition of GTC were characterized using Fourier-transform infrared spectroscopy (FT-IR) and proton nuclear magnetic resonance spectroscopy (¹H NMR). FT-IR spectra were recorded using an IRTracer-100 FT-IR spectrometer (Shimadzu, Japan). For FT-IR analysis, GA, CMC, and GTC samples were prepared using the KBr pellet technique, while NH_2_-TK-NH_2_ was measured using the attenuated total reflectance method.

For ^1^H NMR analysis, GA and NH_2_-TK-NH_2_ were dissolved in deuterated dimethyl sulfoxide (DMSO-D_6_), whereas CMC and GTC were dissolved in deuterium oxide (D_2_O). The ^1^H NMR spectra were acquired using an Avance NEO 600 MHz NMR spectrometer (Bruker, Germany).

### Preparation of sGTND

PEI was incubated with siG6PD at various N/P ratios at room temperature for 30 min to form PEI-siG6PD complexes. The complexes were then loaded into a 1% agarose gel and subjected to electrophoresis at 80 V for 25 min. The gel was imaged using a UV imaging system to determine the N/P ratio at which siG6PD was completely condensed.

The sGTND was prepared using a homogenization/emulsification method. A certain proportion of lecithin, Tween 20, and PFH was added to deionized water containing PEI-siG6PD. The mixture was sonicated using an ultrasonic cell disruptor (UP250, Scientz, China) at 100 W for 5 min in an ice bath, with a cycle of 10 s of operation followed by 10 s of rest. A solution containing CMC and GTC (the ratio of molar masses is 2:1) was then added dropwise, and the mixture was sonicated again for an additional 5 min under the same conditions. After centrifugation at 13,000 rpm for 15 min and washing with PBS, sGTND was obtained. By replacing the GTC-containing solution with a CMC-containing solution in the same procedure, sND could be prepared.

### Characterization of sGTND

The fluorescence intensity of Cy5-labeled siG6PD was measured at Ex/Em = 649 nm/670 nm using a multifunctional microplate reader (Infinite M200pro, Tecan, Switzerland) and a standard curve for Cy5-siG6PD was established. sGTND was prepared using Cy5-siG6PD, and the encapsulation efficiency (EE) of siG6PD was calculated. The morphology and structure of sGTND were observed using a transmission electron microscope (TEM) (JEM-F200, JEOL, Tokyo, Japan), and dynamic light scattering (DLS) (Anton Paar Litesizer 500, Graz, Austria) was employed to measure the polydispersity index, hydrodynamic particle size and surface charge of sGTND.

To verify the serum stability of sGTND, naked siG6PD and sGTND were separately incubated with PBS containing 10% FBS (Gibco, Carlsbad, USA) for 30 min and 24 h. After incubation, the samples were loaded into a 1% agarose gel and subjected to electrophoresis at 80 V for 25 min. The gels were then imaged to assess the stability of sGTND in serum conditions.

To evaluate the long-term stability of the sGTND, the freshly prepared formulations were stored at 4 ℃ for 7 days. Stability was assessed by measuring the particle size at day 0 and day 7 using DLS, with changes in particle size used as the primary indicator. In addition, the samples were visually inspected for phase separation and sedimentation. After gentle shaking to redisperse the sGTND, their general morphology and dispersion state were further examined under an optical microscope.

To assess the ROS responsiveness of sGTND, 1 ml of sGTND solution was centrifuged at 13,000 rpm for 15 min, and the supernatant was discarded. The pellet was resuspended in 1 ml of deionized water with or without 5 mM and 10 mM H₂O₂. The solution was incubated at 37 °C with shaking at 100 rpm. At predetermined time intervals (1, 2, 4, 8, 12, 24, and 48 h), the solution was centrifuged, and the supernatant was collected to measure the release of GA at an absorbance wavelength of 257 nm.

### Cell and animal model

The human hepatocellular carcinoma Huh-7 cells were acquired from ATCC and cultured in Dulbecco’s modified Eagle’s medium (DMEM) containing 10% fetal bovine serum and 1% penicillin/streptomycin at 37℃ in a humidified incubator with 5% CO₂. For all experiments, cells in the logarithmic growth phase were utilized. The cell line was authenticated by short tandem repeat profiling.

Female BALB/c-nude mice, aged 4 weeks, were purchased from Huafukang Biotechnology (Beijing, China). To establish a xenograft tumor model, Huh-7 cells were resuspended in a mixture of PBS and Matrigel at a ratio of 3:1 to a concentration of 5 × 10^7^ cells/ml. Subsequently, 100 µl of this cell suspension was injected into the axilla of each mouse to establish a xenograft tumor model. All animal experiment protocols have been approved by the Experimental Animal Ethics Committee of Qilu Hospital, Shandong University (approval number: DWLL-20250044) and conducted following the National Research Council’s Guide for the Care and Use of Laboratory Animals.

### Intracellular uptake

To evaluate the effects of GA modification and US irradiation on cellular uptake of NDs, cells were divided into the following four groups: (1) sND group; (2) sND + US group; (3) sGTND group; and (4) sGTND + US group. US irradiation was performed at a frequency of 1.0 MHz, an intensity of 0.5 W/cm², and an irradiation time of 60 s. A blank control group was also established.

The Huh-7 cells were seeded into 24-well plates at a density of 5 × 10⁴ cells/ml per well. After the cells had attached to the plate, they were co-incubated with various FITC-labeled NDs. For the US group, US irradiation was applied for 30 min during the co-incubation period. For the GA blocking assay, 1 mL of GA solution (5 µg/mL) was added 0.5 h in advance to saturate the GA receptors on Huh-7 cells prior to the addition of sGTND. In parallel, a normal human hepatocyte cell line (MIHA) was used as a control to compare the targeting specificity of sGTND. Once the total co-incubation time reached 2 h, the cells were fixed and washed. Subsequently, the cells were analyzed using both fluorescence microscopy (Nikon, Tokyo, Japan) and flow cytometry (FCM) (BD FACS Celesta, USA).

### Lysosomal escape

To investigate the ability of siG6PD to escape from lysosomes to the cytoplasm, cells were seeded into confocal dishes and divided into three groups: (1) Naked siG6PD; (2) sGTND; (3) sGTND + US. In each group, siG6PD was labeled with Cy5. After incubation for 4 h, the cells were incubated with serum-free medium containing 100 nM LysoTracker Green at 37℃ for 30 min. Following this, the cells were washed and stained with Hoechst. Observations and photographs were taken using an confocal fluorescence microscope, and quantitative analysis was performed using ImageJ software.

### The *in vivo* biodistribution and tumor targeting

To observe the in vivo biodistribution of sGTND, each tumor-bearing nude mouse was injected via the tail vein with 100 µl of FITC-labeled sGTND (Ex/Em = 488/525 nm). At predetermined time intervals (1 h, 2 h, 4 h, 8 h, and 24 h), the mice were sacrificed, and tumor tissues and major organs were collected. Ex vivo imaging was performed using the IVIS Spectrum imaging system (PerkinElmer, USA).

To evaluate the in vivo tumor-targeting ability of sGTND, tumor-bearing nude mice were randomly divided into two groups (*n* = 3). One group was injected with 100 µl of FITC-sGTND, and the other with FITC-sND. After 24 h, the mice were sacrificed, and tumor tissues and major organs were collected for ex vivo imaging using the IVIS Spectrum imaging system.

### *In vitro* and *in vivo* of CEUI

To investigate the CEUI capability of sGTND, a color doppler US diagnostic device (LOGIQ E9, GE, USA) was employed for evaluation. For the in vitro assessment of US imaging capability, a test model was constructed using a pasteur pipette. A total of 3 ml of either PBS or sGTND solution was added to the pasteur pipette model, which was then immersed in ultrapure water. US treatment was performed using the same parameters, followed by contrast-enhanced imaging. The specific US parameters used were as follows: a 9 L linear transducer with a center frequency of 9.0 MHz, a mechanical index (MI) of 0.8, and a dynamic range of 60 dB.

To evaluate the in vivo CEUI capability, a tumor xenograft model in nude mice was established. After anesthetizing the nude mice, 100 µl of either PBS or sGTND was injected via the tail vein. Contrast-enhanced imaging of the tumor site was then performed using a center frequency of 9.0 MHz, a mechanical index (MI) of 0.5, and a dynamic range of 48 dB.

### Hemolysis assay

To demonstrate the in vivo biocompatibility of GTND, a hemolysis assay was conducted. PBS was used as the negative control, and deionized water was used as the positive control. Various concentrations of GTND (1000 µg/ml, 500 µg/ml, 250 µg/ml, 125 µg/ml, 62.5 µg/ml, and 31.25 µg/ml) were each mixed uniformly with a 4% red blood cell suspension. After incubation at room temperature for 2 h, the mixtures were centrifuged to collect the supernatants. The absorbance of each group was measured at 542 nm using a microplate reader. The hemolysis rate (%) was calculated as follows: Hemolysis rate (%) = (Experimental group OD value - Negative control group OD value) / (Positive control group OD value - Negative control group OD value)× 100%.

### Intracellular ROS levels

Cells were divided into the following four groups: (1) sND; (2) sND + US; (3) sGTND; (4) sGTND + US, with an additional blank control group. Cells were seeded in 6-well plates at a density of 6 × 10⁵/ml per well. After the cells adhered to the plates, they were subjected to the respective treatments for 2 h. The culture medium was then removed, and the cells were incubated with the DCFH-DA probe diluted 1:1000 in serum-free medium at 37℃ for 30 min, imaging was performed using fluorescence microscopy. In a manner similar to the aforementioned procedures, following digestion and washing, the cells were collected and analyzed using FCM.

### Western blot

Huh-7 cells were seeded in a 6-well plate at a density of 6 × 10⁵/ml per well. After the cells adhered, they were subjected to different treatments. After 48 h of culture, the cells were lysed on ice for 30 min using RIPA lysis buffer containing PMSF. The lysates were centrifuged, and the supernatants containing the proteins were collected. The proteins were mixed with loading buffer and boiled. After separation by SDS-PAGE electrophoresis, the proteins were transferred to PVDF membranes. The membrans were blocked with 5% skim milk at room temperature and then incubated sequentially with primary and secondary antibodies. After adding the chemiluminescent substrate, the membranes were imaged using a chemiluminescence imaging system (Tanon, Shanghai, China) .

### *In vitro* gene silencing efficiency

Huh-7 cells were seeded in a 6-well plate at a density of 6 × 10⁵/ml per well. After overnight adherence, the cells were subjected to different treatments: (1) PBS; (2) Lipo3000; (3) sND; (4) sND + US; (5) sGTND; (6) sGTND + US. For the Lipo3000 group (containing 5 µl of 50nM siG6PD), the cells were treated in DMEM medium containing 10% FBS without penicillin-streptomycin solution. After 6 h, the medium was replaced, and the cells were cultured for another 48 h. Total protein was then extracted, and the efficiency of in vitro gene silencing was determined by western blot.

### Immunofluorescence

The effect of sGTND combined with UTMD on the intracellular localization of NRF2 was verified by immunofluorescence. Cells were divided into the following six groups: (1) PBS; (2) GA; (3) sND; (4) sND + US; (5) sGTND; and (6) sGTND + US. Huh-7 cells were seeded in 24-well plates at a density of 5 × 10⁴ cells/ml per well. After the cells had attached to the plate, they were treated with the respective agents. After a 48-h incubation period, the cells were subjected to fixation, permeabilization, blocking, and incubation with primary and secondary antibodies, followed by Hoechst staining. Finally, the cells were imaged using fluorescence microscope.

### NRF2, xCT/SLC7A11, ME1 and IDH1 expression levels

Huh-7 cells were seeded in a 6-well plate at a density of 6 × 10⁵/ml per well. After overnight adherence, the cells were subjected to the following treatments: (1) PBS; (2) GA; (3) sND; (4) sND + US; (5) sGTND; (6) sGTND + US. After 48 h of culture, total protein was extracted. The protein levels of NRF2, xCT/SLC7A11, ME1 and IDH1 were determined using western blot.

### NADP+/NADPH levels

Huh-7 cells were seeded in a 6-well plate at a density of 6 × 10⁵/ml per well. After overnight incubation for adherence, the cells were subjected to different treatments: (1) PBS; (2) GTND + US; (3) sND; (4) sND + US; (5) sGTND; (6) sGTND + US. After 48 h of culture, the cells were digested and collected for cell counting. The cells were then lysed on ice, and the lysates were centrifuged to collect the supernatants. The intracellular levels of NADP+/NADPH were measured using an NADP+/NADPH assay kit according to the manufacturer’s instructions.

### The levels of TCA cycle metabolites

Cells were divided into following six groups: (1) PBS; (2) GTND + US; (3) sND; (4) sND + US; (5) sGTND; (6) sGTND + US. Huh-7 cells were seeded in a 6-well plate at a density of 6 × 10⁵/ml per well. After overnight incubation to allow adherence, the cells were treated as described above for 48 h. The cells were then digested and collected for cell counting. The cells were lysed on ice, and the lysates were centrifuged to collect the supernatants. The levels of malate, isocitrate, and fumarate in the cells were measured using malate, isocitrate, and fumarate content assay kits according to the manufacturer’s instructions.

### *In vitro* antitumor efficacy

The cell experiments were divided into the following groups: (1) PBS; (2) GTND + US; (3) sND; (4) sND + US; (5) sGTND; (6) sGTND + US. Cell viability was determined using the CCK8 assay. Huh-7 cells were collected after digestion and diluted to a concentration of 5 × 10⁴/ml. The cell suspension was mixed thoroughly, and 100 µl was seeded into each well of a 96-well plate. After overnight adherence, the cells were treated with the above-mentioned. After 48 h of incubation, the cells were washed with PBS, and the drug-containing culture medium was replaced with a culture medium containing 10% CCK8. The cells were then incubated at 37℃. The absorbance of each well at 450 nm was measured using a microplate reader.

The proliferative capacity of cells was verified by the EDU assay. Cells were seeded into 96-well plates and, after attachment, were subjected to the respective treatments. Following a 48-h incubation period, the cells were processed in accordance with the operational steps outlined in the Edu-594 Cell Proliferation Kit. Subsequently, the cells were observed under a fluorescence microscope.

The wound healing assay was used to assess cell migration ability. Three evenly spaced straight lines were drawn on the back of a 6-well plate using a marker. Huh-7 cells were seeded in the 6-well plate at a density of 1 × 10⁶/ml per well. When the cell density reached over 90%, a 200 µl pipette tip was used to draw straight lines perpendicular to the bottom of the 6-well plate. The cells were washed three times with PBS and then replaced with DMEM culture medium containing 2% FBS. The cells were treated as described above and cultured for an additional 48 h. Photos were taken at the same positions at 0 h and 48 h. The migration distance of the cells was calculated using ImageJ. The cell migration rate was calculated using the following formula: Cell migration rate (%) = (0 h average scratch area − 48 h average scratch area) / 48 h average scratch area×100%.

The apoptosis of cells was detected using a Annexin V-FITC and PI assay. The Huh-7 cells were seeded into six-well plates at a density of 6 × 10⁵ cells/ml per well. After overnight attachment, the cells were subjected to various treatments. Following a 48-hour incubation period, the cells were digested and collected. After washing, the cells were resuspended in binding buffer and stained with Annexin V-FITC and PI. Subsequently, the cells were analyzed by FCM.

### *In vivo* antitumor efficacy

When the tumor volume reached 50 mm³, tumor-bearing nude mice were randomly divided into six groups (*n* = 5): (1) PBS; (2) GTND + US; (3) sND; (4) sND + US; (5) sGTND; (6) sGTND + US. The drugs were administered via tail vein injection. The dosing concentrations were as follows: GA at 2 mg/kg and siG6PD at 6 µg/kg. The US irradiation conditions were set at 1.25 W/cm², 60 s, and 1 MHz. The treatment was administered every 3 days for a total of 4 treatments. The tumor volume and body weight of the mice were monitored every 2 days. The tumor volume was calculated using the formula: Tumor volume = 0.5 × length × width^2^. At the end of the experiment, the mice were sacrificed, and the tumor tissues were removed for photography, weighing, and fixation. Tumor growth inhibition(%) = 100% × (1-mean tumor weight of experimental group/mean tumor weight of control group). The tumor tissues were subjected to H&E, Ki67, and TUNEL staining, and the protein levels of NRF2, xCT/SLC7A11, ME1, and IDH1 were further determined by immunohistochemical analysis. Blood samples were collected from both the control group and the sGTND-treated group of nude mice. Subsequently, various serum markers were analyzed, including alanine aminotransferase (ALT), aspartate aminotransferase (AST), blood urea nitrogen (BUN), creatinine (CREA), and uric acid (UA). Major organs from the mice were also removed, fixed, and stained with H&E.

### Statistical analysis

All data are expressed as mean ± standard deviation (mean ± SD). Statistical analysises were performed using GraphPad Prism 9.5.1. Comparisons between two groups were analyzed using Student’s t-test, while differences among multiple groups were analyzed using one-way analysis of variance (ANOVA). *P* < 0.05 was considered indicate statistical significance.

## Results and discussion

### Synthesis and characterization of the GTC polymer

**Fig. 2:**
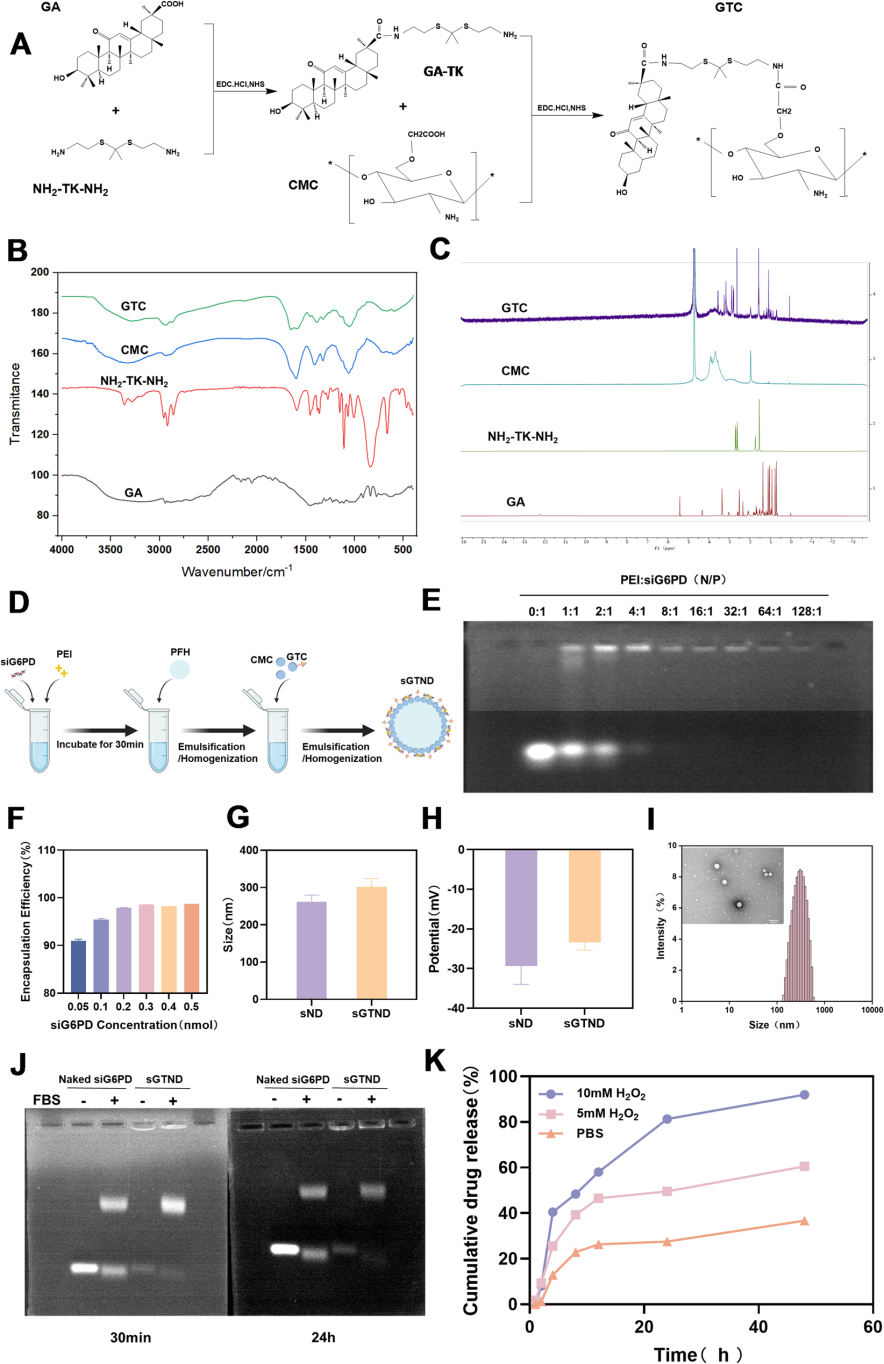
Synthesis and characterization of the GTC and sGTND. (**A**) Schematic diagram illustrating the synthesis of the GTC polymer from the raw materials GA, CMC and NH_2_-TK-NH_2_. (**B**) FT-IR spectrum of GTC, CMC, NH_2_-TK-NH_2_ and GA. (**C**) ¹H NMR spectra of GTC, CMC, NH_2_-TK-NH_2_ and GA. (**D**) Synthesis of sGTND via a homogenization/emulsification method. (**E**) Adsorption efficiency of PEI for siG6PD at different N/P ratios evaluated by agarose gel electrophoresis. (**F**) Encapsulation efficiency of PEI-siG6PD prepared with different amounts of siG6PD. (**G**) Particle size of sND and sGTND. (**H**) Zeta potential of sND and sGTND. (**I**) TEM images and particle size distribution of sGTND. Scale bar: 500 nm. (**J**) Serum stability of sGTND evaluated by agarose gel electrophoresis. (**K**) ROS-responsive GA release of sGTND over 48 h in different media (*n* = 3)

CMC is recognized as a versatile biomaterial for DDS due to its excellent biocompatibility, biodegradability, non-toxicity, and inherent antitumor activity. Moreover, the abundant amino and carboxyl groups of CMC facilitate various chemical modifications, enabling efficient drug-graft or targeted delivery [25, 26]. In this study, with a molecular weight of 318 kDa (Figure S1) was employed as the scaffold material, and GA was conjugated via a TK linker to obtain the GTC polymer. This design enabled both efficient GA loading and ROS-responsiveness. As illustrated in Fig. 2A, GTC polymer is synthesized via a two-step amidation reaction, GA-TK intermediate formation in DMSO, followed by CMC conjugation in water. The detailed synthetic procedure is provided in Sect. "Synthesis and characterization of GTC polymer". of the Materials and Methods. The chemical structure of the GTC polymer was characterized using FT-IR and ¹H-NMR. In the FT-IR spectrum of the GTC polymer (Fig. 2B), compared with that of CMC, characteristic peaks of amide bonds appeared at 1654 cm⁻¹ and 1592 cm⁻¹, indicating that amidation had occurred in CMC. In the ¹H-NMR spectrum of the GTC polymer (Fig. 2C), compared with that of CMC, characteristic proton peaks of the GA methyl, methylene, and methine groups appeared at 1.0 ppm, and the characteristic peak of the -SC(CH₃)₂S- group in NH₂-TK-NH₂ appeared at 1.5 ppm. What’s more, in the FT-IR and ^1^H-NMR spectra of GTC, in addition to confirming the presence of GA and NH_2_-TK-NH_2_, the retained C-O stretching vibrations around 1000–1200 cm^− 1^ in FT-IR and the preserved sugar ring protons at 3.0-4.5 ppm in ^1^H-NMR also verified the preservation of the CMC backbone structure. The above results collectively demonstrate that GA and NH_2_-TK-NH_2_ were successfully grafted onto CMC through amide bond formation. The grafting efficiency of GA was determined to be 53.6%.

### Preparation and characterization of sGTND

The sGTND was prepared using a homogenization/emulsification method [27] (Fig. 2D). PEI is a cationic transfection reagent that can complex with negatively charged siRNA and facilitate the escape of siRNA from lysosomes through the proton sponge effect [28]. Agarose gel electrophoresis results (Fig. 2E and Figure S7) showed that when the N/P ratio of PEI to siG6PD was 8:1, siG6PD was completely adsorbed. Therefore, an N/P ratio of 8:1 was chosen for subsequent experiments.

To determine the optimal EE of PEI-siG6PD, different amounts of siG6PD (0.05 nmol, 0.1 nmol, 0.2 nmol, 0.3 nmol, 0.4 nmol, and 0.5 nmol) were tested. As shown in Fig. 2F, the EE gradually increased with the increase of siG6PD loading from 0.05 to 0.3 nmol. When the siG6PD loading reached 0.3 nmol and above, the EE stabilized at over 98%. Therefore, a siG6PD loading of 0.3 nmol was selected for further studies.

To investigate the effect of GA targeting modification on the physicochemical properties of the NDs, the particle size and zeta potential of both sND (non-targeted NDs) and sGTND (targeted NDs) were measured (Fig. 2G and H). The average particle size of sGTND increased to 301.7 ± 22.11 nm compared to 261.4 ± 18.21 nm for sND. Both sND and sGTND exhibited negative zeta potentials (-23.43 ± 1.890 mV for sGTND and − 29.37 ± 4.631 mV for sND). The less negative zeta potential of sGTND compared to sND was attributed to the negatively charged CMC in the formulation [29], while GA is a neutral small molecule ligand [30]. The negative charge of sGTND not only prolongs its circulation time in vivo but also reduces its accumulation in lysosomes through electrostatic repulsion with the negatively charged lysosomal membrane, thereby enhancing the release efficiency of the encapsulated drug or gene [31]. The morphology and structure of sGTND were observed by TEM (Fig. 2I). sGTND exhibited a regular spherical shape with a size of around 300 nm, uniform dispersion, and a distinct core-shell structure.

The long-term stability for sGTND was investigated by measuring the particle size variation of the prepared sGTND over a period of 7 days. The mean particle size of sGTND after 7 days was 324.5 ± 32.11 nm (Figure S2B), which showed no statistically significant difference compared with that on day 0 (301.7 ± 22.11 nm) (Figure S2C). Minor phase separation and sedimentation were observed after 7 days of storage, however, the suspension became homogeneous upon gentle shaking. Optical microscopy revealed that the nanodroplets maintained good dispersion (Figure S2A). These results indicate that sGTND could be stably stored at 4℃ for at least 7 days.

The inherent susceptibility of naked siRNA to nuclease degradation in serum leads to short half-life and rapid clearance, limiting its clinical therapeutic applications [32]. To investigate the stability of siG6PD in sGTND, a serum stability experiment was conducted. As shown in Fig. 2J, siG6PD encapsulated in sGTND could not migrate in agarose gel, whereas naked siG6PD could. When incubated with 10% FBS, naked siG6PD was degraded within 30 min, while siG6PD in sGTND remained stable for up to 24 h. This indicates that sGTND effectively protects siG6PD from enzymatic degradation.

The loading efficiency of GA in sGTND was 18.5%. To confirm the ROS-responsive drug release ability of sGTND, the cumulative release of GA from sGTND in different media was studied, and the results are shown in Fig. 2K. In PBS, the release rate of GA was 26% at 24 h and 36% at 48 h. In 5 mM H_2_O_2_, the release rate of GA was 40% at 8 h, 49% at 24 h and 60% at 48 h. When the H_2_O_2_ concentration was increased to 10 mM, the release rate of GA was 58% at 12 h, 81% at 24 h, and 91% at 48 h. These results demonstrate that sGTND can release GA in response to ROS in a concentration-dependent manner, with higher ROS concentrations facilitating more sustained and complete drug release.

### Intracellular uptake, lysosomal escape and biodistribution of sGTND

**Fig. 3:**
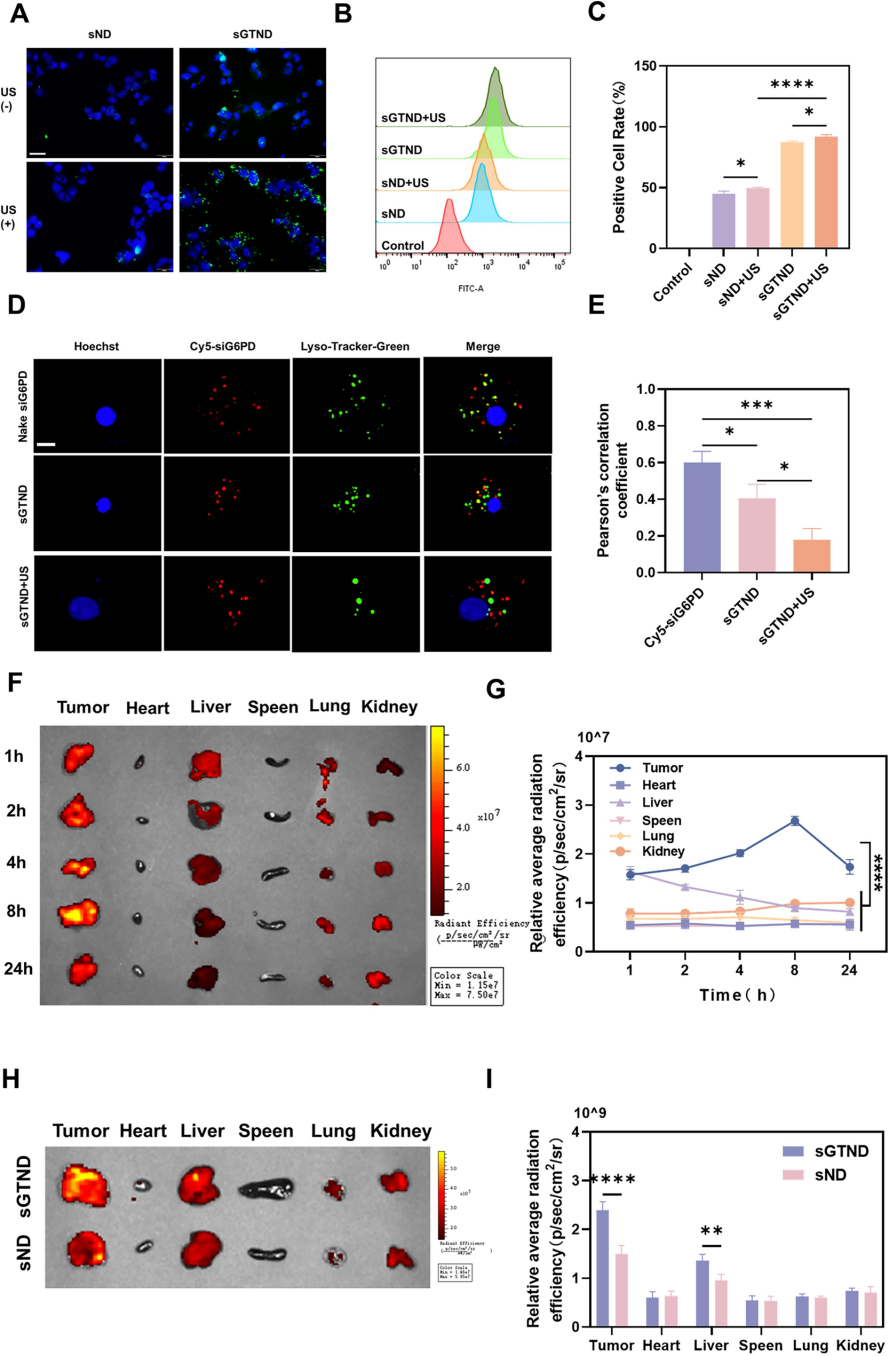
Intracellular uptake, lysosomal escape and biodistribution of sGTND. (**A**) Fluorescence images showing the uptake of FITC-labeled sND and sGTND by Huh-7 cells with or without US treatment. Scale bar: 50 μm. (**B**) FCM analysis of the uptake of FITC-labeled sND and sGTND by Huh-7 cells with or without US treatment. (**C**) Percentage of positive cells for uptake of NDs in different treatment obtained from B. (**D**) Colocalization of Cy5-siG6PD (red) with lysosomes (green) under different treatments. Scale bar: 10 μm. (**E**) Pearson’s correlation coefficients analyzed using ImageJ based on the data from D. (**F**) In vivo biodistribution of FITC-sGTND in major organs (tumor, heart, liver, spleen, lung and kidney) at various time points. (**G**) Quantitative analysis of the average radiative efficiency from D. (**H**) In vivo biodistribution of FITC-sGTND and sND at 24 h. (**I**) Quantitative analysis of the average radiative efficiency from H. All results are presented as mean ± SD (*n* = 3). *p < 0.05, **p < 0.01, ***p < 0.001 and ****p < 0.0001

GA serves as a targeting ligand for HCC and, when conjugated to NDs, enhances their active targeting to cancer cells [33]. UTMD increases the passive cellular uptake of NDs by enhancing membrane permeability via sonoporation [34]. As shown in Fig. 3A, cells treated with sGTND + US exhibited stronger green fluorescence compared to other groups. FCM results indicated that the positive cell ratio in the sGTND + US group was 1.05 times higher than that in the sGTND group and 1.86 times higher than that in the sND + US group (Fig. 3B and C). These findings demonstrate that the active targeting of GA-modified sGTND, in combination with the sonoporation effect mediated by UTMD, significantly enhances cellular uptake of the NDs.

The function of GA modification on sGTND was further investigated. As shown in Figure S3A, Huh-7 cells pretreated with GA exhibited markedly reduced uptake of sGTND, as evidenced by the significantly lower green fluorescence intensity in the sGTND + GA group compared with the sGTND group. FCM analysis (Figure S3B and S3C) further revealed that the average fluorescence intensity in the sGTND + GA group was reduced by 74.6% relative to that in the sGTND group. According to previous reports, although normal hepatocytes also express a certain level of GA receptors, the amount of GA receptors on the surface of HCC cells is significantly higher than that on normal hepatocytes [35]. Therefore, we compared the binding of sGTND to normal human hepatocytes (MIHA) and to HCC cells (Huh-7). As shown in Figure S4A, the green fluorescence associated with sGTND binding was markedly weaker in MIHA cells than in Huh-7 cells. Consistently, FCM analysis (Figures S4B and S4C) revealed that the average fluorescence intensity of the Huh-7 + sGTND group was 5.5-fold higher than that of the MIHA + sGTND group. These results suggest that GA-modified sGTND can specifically recognize HCC cells via GA-receptor interactions.

Free siRNA is prone to being captured and degraded by lysosomes, resulting in relatively low efficiency in silencing target genes. As a cationic polymer, PEI can adsorb siRNA. Moreover, due to its proton sponge effect, PEI can induce lysosomal rupture and release siRNA [36]. As shown in Figs. 3D and E, in the Naked siG6PD group, the red fluorescence from Cy5-siG6PD colocalized with the green fluorescence of lysosomes at 4 h, producing a distinct yellow fluorescence with a Pearson correlation coefficient of 0.60, indicating that free siG6PD failed to escape from lysosomes. In the sGTND group, the yellow fluorescence resulting from the colocalization of Cy5-siG6PD and lysosomes was significantly reduced, with a Pearson correlation coefficient of 0.41. In contrast, in the sGTND + US group, almost no yellow fluorescence was observed, and the green and red fluorescence were clearly distinguishable, with a Pearson correlation coefficient of 0.18. This is likely because the generation of ROS including singlet oxygen (^1^O_2_) under the action of UTMD disrupted the lysosomal membrane [37], thereby facilitating the lysosomal escape of siG6PD. Therefore, the combination of sGTND and US effectively promotes the escape of siG6PD from lysosomal into the cytoplasm, enabling it to exert its gene-silencing effect.

The in vivo biodistribution and targeting of sGTND were further investigated using the IVIS spectrum imaging system. As shown in Fig. 3F and G, fluorescence signals were primarily localized in tumor tissues, likely due to the enhanced permeability and retention effect and the active targeting of sGTND. Fluorescence signals were detectable in tumor tissues as early as 1 h post-injection, peaked at 8 h, and remained significant at 24 h, indicating prolonged retention of sGTND within the tumor. In vivo small-animal fluorescence imaging was also performed, and the overall trend was consistent with the ex vivo imaging results (Figure S5). Additionally, due to the phagocytic activity of the reticuloendothelial system in the liver and the low expression of GA receptors on normal hepatocytes [38, 39], higher fluorescence signals were observed in the liver among the major organs, peaking at 1 h and gradually decreasing over time.

The in vivo targeting efficacy of sGTND and sND is shown in Fig. 3H and I. At 24 h post-injection, the fluorescence intensity in tumor tissues was significantly higher in the sGTND group than in the sND group, demonstrating superior targeting ability of sGTND.

### Biocompatibility evaluation and CEUI ability

**Fig. 4:**
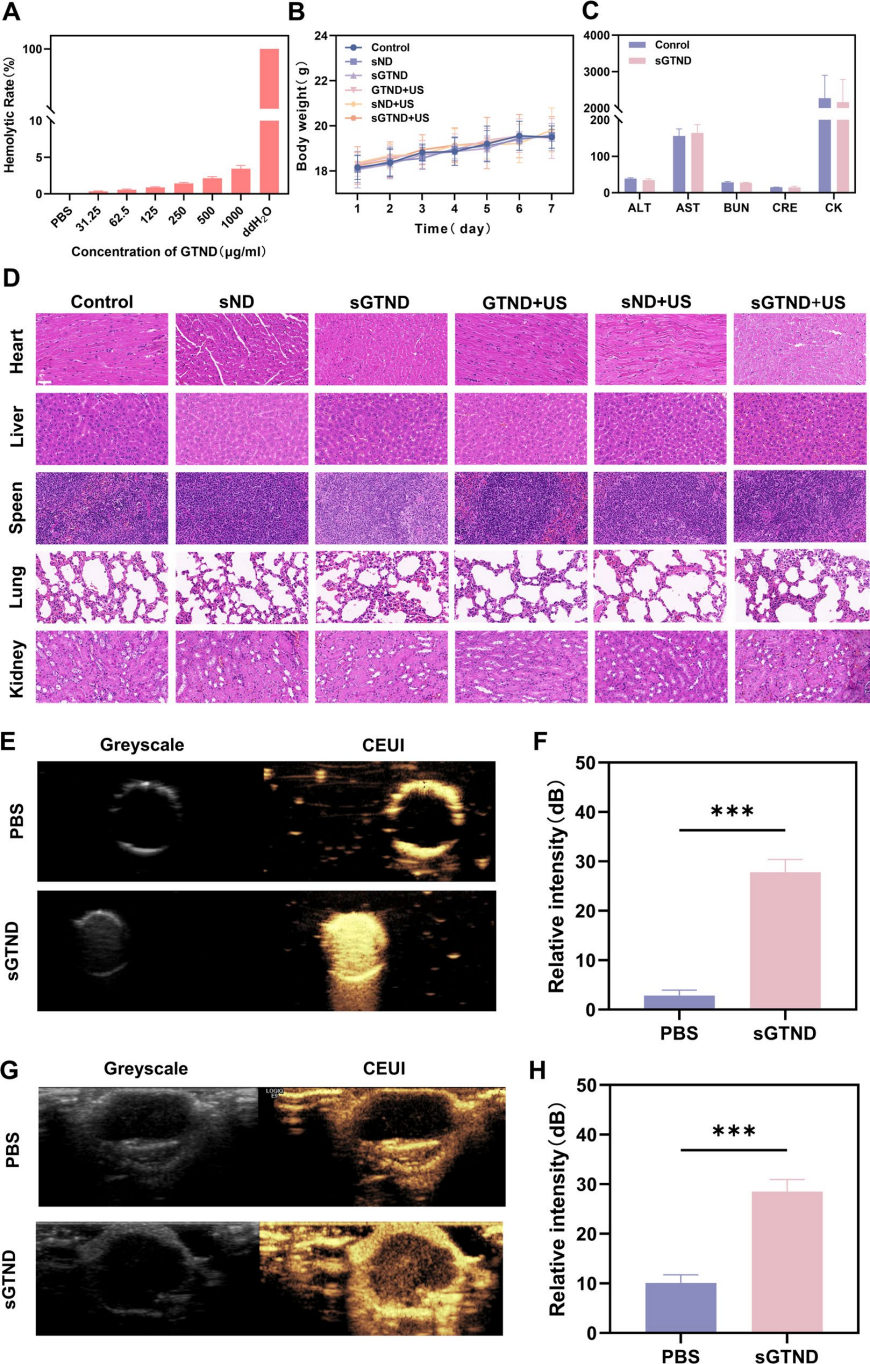
Biocompatibility evaluation and CEUI ability. (**A**) Hemolysis rate of GTND at concentrations ranging from 31.25 µg/mL to 1000 µg/mL, with PBS as the negative control and ddH_2_O as the positive control. (**B**) Body weight change curves of Huh-7 tumor-bearing mice in different treatment groups within 12 days. (**C**) Biochemical blood analysis of nude mice in the control and sGTND groups, including ALT, AST, BUN, CRE and CK levels. (**D**) H&E staining of hearts, livers, spleens, lungs and kidneys of nude mice in different treatment groups. Scale bar: 50 μm. (**E**) In vitro CEUI imaging of sGTND. (**F**) Relative contrast intensity from E. (**G**) In vivo CEUI imaging of sGTND. (**H**) Relative contrast intensity from G. All results are presented as mean ± SD (*n* = 3). *** *p* < 0.001 and ****p < 0.0001

As shown in Fig. 4A, the hemolysis rate remains below 5% even when the GTND concentration reaches as high as 1000 µg/ml. After the completion of the treatment, the survival rate of mice in all groups was 100%. During the treatment process, no significant adverse reactions were observed in the mice of any group, and there were no significant differences in the body weight changes of the mice (Fig. 4B). The blood biochemical indices, including ALT, AST, CK, BUN, and CRE, for the control group and the sGTND group were all within the reference range, and no statistically significant differences were observed between the two groups (Fig. 4C). The H&E staining of the vital organs (heart, liver, spleen, lung, and kidney) of mice in all groups showed no significant pathological changes (Fig. 4D). In conclusion, the NDs exhibit excellent biocompatibility.

To evaluate the CEUI ability of sGTND under US irradiation, both in vivo and in vitro US imaging were performed using grayscale and CEUI modes. As shown in Fig. 4E and F, in the in vitro experiments, the sGTND group exhibited significant echo enhancement in CEUI mode compared to the PBS group after US irradiation. In the in vivo experiments (Fig. 4G and H), the tumor sites in mice from the PBS group showed minimal echo signals, whereas the tumor sites in the sGTND group exhibited markedly enhanced echo signals, with clear visualization of tumor size and margins. The pronounced increase in echo intensity of sGTND under US irradiation is attributed to the presence of PFH. US can induce the liquid-gas phase transition of PFH, thereby enhancing its contrast with the surrounding tissues and facilitating diagnostic assistance [40, 41]. Therefore, sGTND can serve as an effective US contrast agent for the visualization, controllability, and precise treatment of tumors.

### The *in vitro* silencing efficiency, ROS generation, and activation of the NRF2 pathway by sGTND in combination with UTMD

**Fig. 5:**
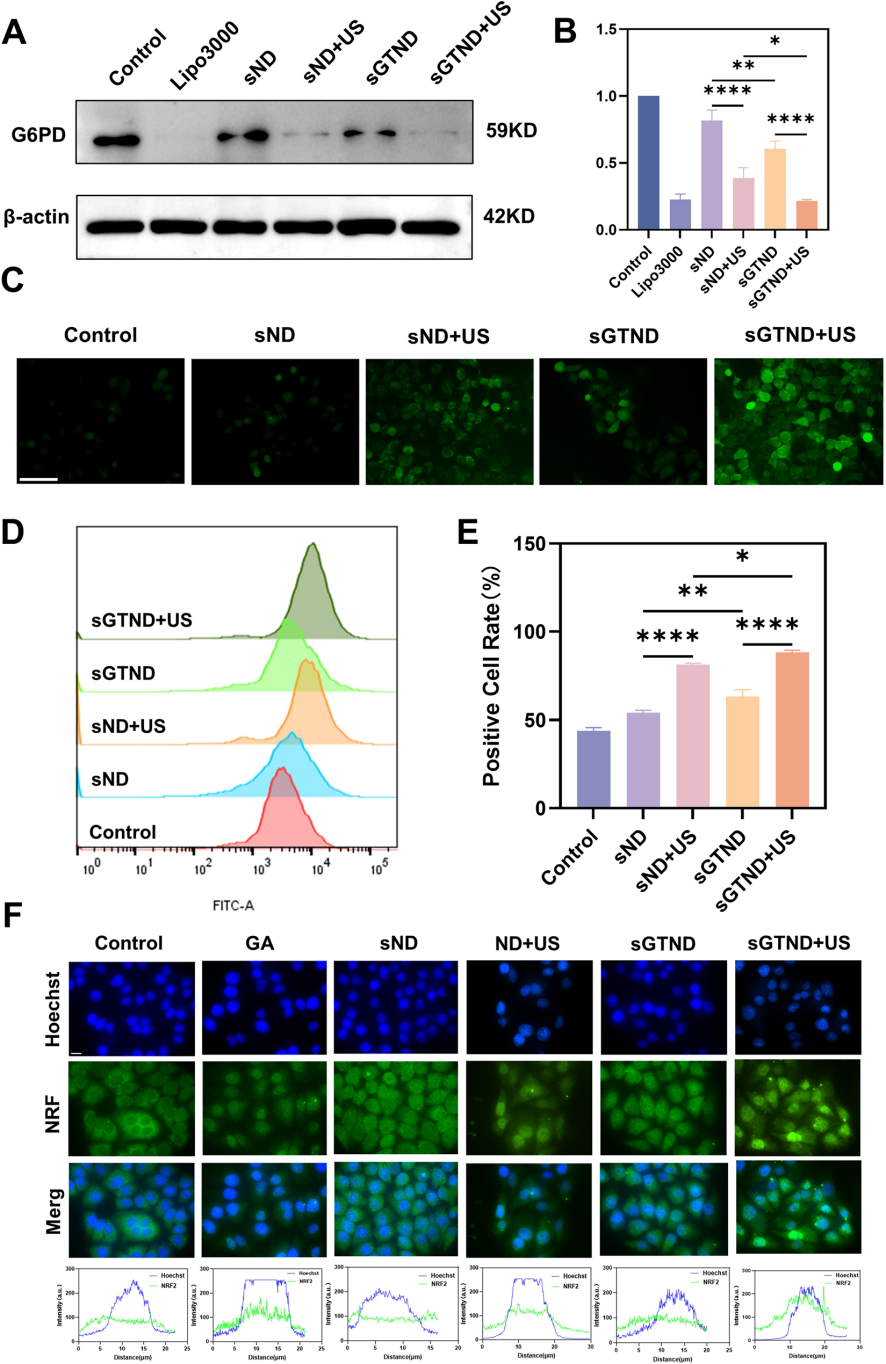
The in vitro silencing efficiency, ROS generation, and activation of the NRF2 pathway by sGTND in combination with UTMD. (**A**) Western blot analysis of G6PD expression in Huh-7 cells under different treatments. (**B**) Quantitative analysis of G6PD under different treatments from A. (**C**) Fluorescence images of intracellular ROS in Huh-7 cells under different treatments through DCFH-DA staining. Scale bar: 50 μm. (**D**) FCM analysis of intracellular ROS in Huh-7 cells under different treatments. (**E**) Percentage of ROS-positive cells from D. (**F**) Immunofluorescence images of NRF2 (green) in Huh-7 cells under different treatments, scale bar:10 μm. All data are presented as mean ± SD (*n* = 3). ** *p* < 0.01 and ****p < 0.0001

Using Lipo3000 as a positive control, western blot was employed to determine the efficiency of gene silencing in vitro under different treatments (Fig. 5A and Figure S8). Quantitative analysis showed that the expression levels of G6PD were reduced in all treated groups (Fig. 5B). Among them, the sGTND + US group exhibited the highest gene silencing efficiency, reaching 78%, which was comparable to that of the Lipo3000 group. This may be attributed to the fact that both GA-targeted modification and UTMD can enhance cellular uptake of NDs. These results indicate that the combination of sGTND and UTMD possesses excellent in vitro gene silencing capability.

In our previous studies, UTMD effects generated by US acting on certain US-responsive NDs have been proven to induce an increase in intracellular ROS levels [21–23]. For the sGTNDs prepared in this study, intracellular ROS levels were also detected using the DCFH-DA probe. Fluorescence microscopy results revealed that only weak green fluorescence was observed in the sND and sGTND groups, while the green fluorescence signals in the sND + US and sGTND + US groups were significantly enhanced, with the sGTND + US group showing the strongest signal (Fig. 5C). The results from FCM were consistent with those from fluorescence microscopy (Figs. 5D and E). These findings suggest that UTMD effects generated by US acting on sGTNDs can induce high levels of ROS generation. This elevated ROS level can further facilitate ROS-responsive GA release, ROS-mediated NRF2 activation and lysosomal membrane disruption for lysosomal escape.

NRF2 activation is marked by enhanced expression and cytoplasm-to-nucleus translocation. GA has been demonstrated to promote NRF2 dissociation from KEAP1, thereby upregulating NRF2 expression and facilitating its nuclear translocation [15]. This phenomenon was confirmed by immunofluorescence analysis of the GA group in Fig. 5F, which showed increased green fluorescence intensity and enhanced nuclear accumulation of green fluorescence. Interestingly, the ND + US group exhibited similar fluorescence patterns suggesting NRF2 activation, despite the absence of GA. The mechanism explaining this phenomenon is that UTMD can elevate ROS levels. Studies have shown that under elevated cellular ROS levels, ROS react with cysteine residues within the KEAP1 protein, leading to conformational changes in KEAP1 and subsequent release of NRF2 from the KEAP1-CUL3-RBX1 complex [42, 43]. As expected, due to the dual effects of GA and UTMD, the sGTND + US group exhibited stronger nuclear and overall fluorescence intensity compared to the GA and ND + US groups, suggesting a more pronounced promotion of NRF2 nuclear translocation and upregulation of total expression. In contrast, the fluorescence in the control, sND, and sGTND groups was weak and primarily localized in the cytoplasm, indicating that NRF2 activation did not occur.

### The mechanism of sGTND combined with UTMD in inhibiting TCA

NADPH in cells is generated through three pathways: PPP, ME1 and IDH1, with PPP being the primary source of NADPH [11]. Studies have demonstrated that tumors with activated NRF2 pathway consume NADPH through upregulation of xCT/SLC7A11, causing these tumors to rely on the PPP pathway to counteract oxidative stress [10]. Therefore, we hypothesized that under conditions of NRF2 activation, simultaneous inhibition of G6PD, the key rate-limiting enzyme of PPP, would force tumors to depend on ME1 and IDH1 to maintain intracellular NADPH levels. This metabolic reprogramming would lead to the efflux of TCA cycle substrates (malate and isocitrate), ultimately resulting in TCA cycle disruption. Subsequently, we analyzed the proteins and substrates involved in these metabolic processes.

**Fig. 6:**
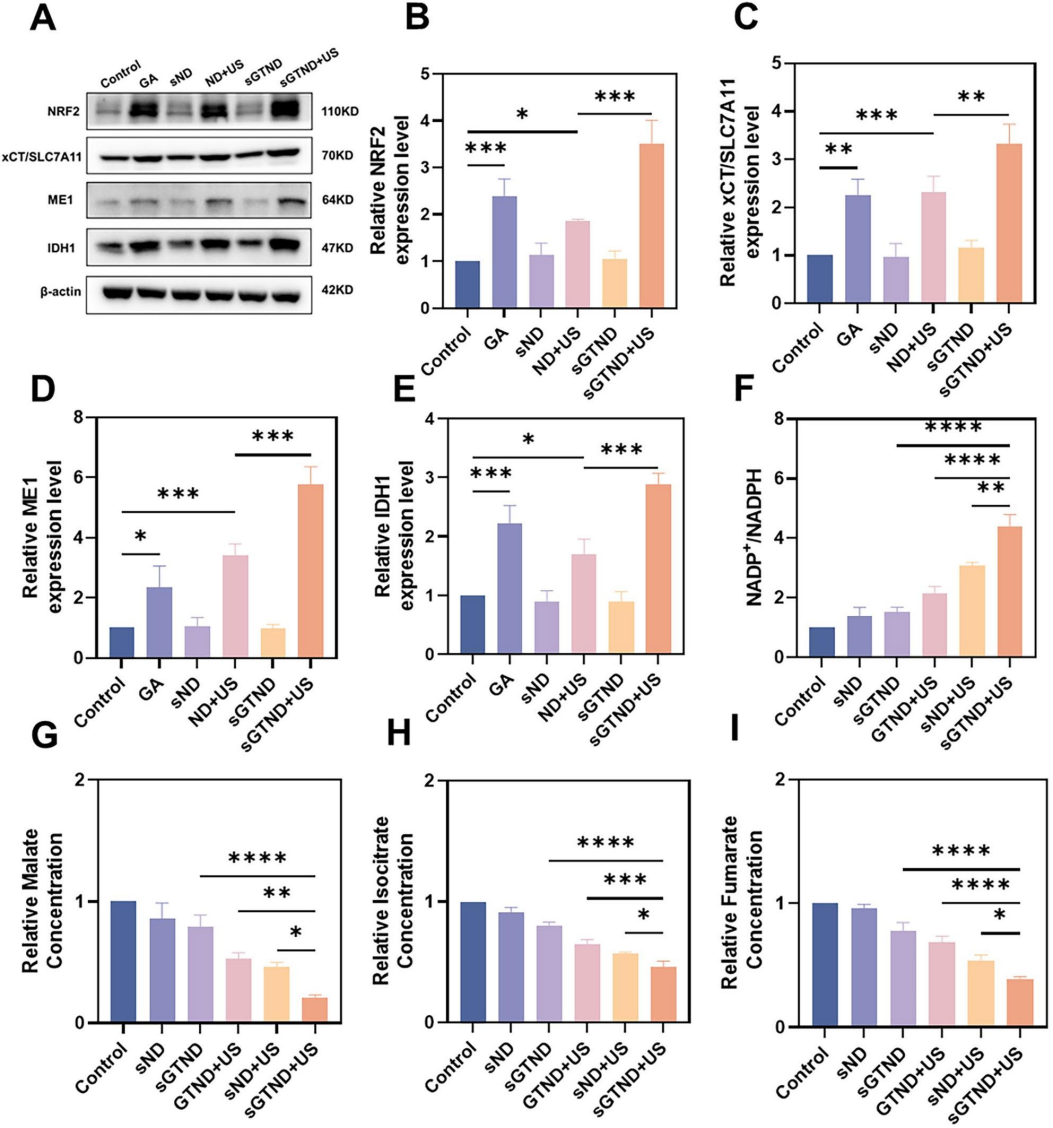
The mechanism of sGTND combined with UTMD in inhibiting TCA. (**A**) Western blot analysis of NRF2, xCT/SLC7A11, ME1, and IDH1 in Huh-7 cells under different treatments. (B-E) Quantitative analysis of NRF2 (**B**), xCT/SLC7A11 (**C**), ME1 (**D**) and IDH1 (**E**) expression from A. (**F-I**) Levels of NADP+/NADPH (**F**), malate (**G**), isocitrate (**H**) and fumarate (**I**) in Huh-7 cells under different treatments. All results are expressed as mean ± SD (*n* = 3). *p < 0.05, **p < 0.01, ***p < 0.001 and ****p < 0.0001

NRF2, xCT/SLC7A11, and the TCA cycle-related markers ME1 and IDH1 were detected by western blot (Fig. 6A and Figure S9). NRF2 expression (Figs. 6A and B) revealed a trend consistent with the immunofluorescence results shown in Fig. 5F, further confirming that sGTND combined with UTMD can significantly activate NRF2. Activation of the NRF2 pathway can lead to increased expression of xCT/SLC7A11 (Fig. 6A and C), thereby consuming NADPH. Concurrently, NRF2 activation also enhances the expression of ME1 (Fig. 6A and D) and IDH1 (Fig. 6A and E), with the most significant elevation of ME1 and IDH1 expression occurring under combined NRF2 activation and G6PD inhibition conditions (sGTND + US group). These findings provide preliminary support for our proposed hypothesis at the protein expression level.

All the aforementioned protein changes are aimed at maintaining intracellular NADPH levels. Given that the NADP+/NADPH ratio serves as an important indicator reflecting cellular NADPH levels, we employed the WST-8 method to detect the NADP+/NADPH ratio in each treatment group. As shown in Fig. 6F, the sND + US and sGTND + US groups, which had activated the NRF2 pathway with further reduced G6PD expression, exhibited higher NADP+/NADPH ratios compared to the GTND + US group without G6PD inhibition. Compared with the sND + US group, the sGTND + US group had higher intracellular NADP+/NADPH levels, which may be due to the combined effect of GA release and targeting modification. Since the sND group only reduced the expression of G6PD, the changes in intracellular NADP+/NADPH levels were not significant compared with the control group. These results indicate that the combination of sGTND and UTMD can consume intracellular NADPH and increase the NADP+/NADPH levels.

Subsequently, intracellular levels of malate, isocitrate and fumarate were measured to validate the inhibitory effect of combined sGTND and UTMD treatment on TCA cycle activity. As shown in Fig. 6G, compared with the control group, the sND groups only reduced the expression of G6PD, and the changes in intracellular malate content were not significant. The GTND + US group, which had activated the NRF2 pathway, showed a decrease in intracellular malate content. The sND + US and sGTND + US groups, which had activated the NRF2 pathway and inhibited the expression of G6PD, exhibited further reductions in malate content. Besides, compared with the sND + US group, the sGTND + US group had lower intracellular malate content, which was consistent with the changes in intracellular NADP+/NADPH levels. This can be attributed to the targeting and NRF2 activation effect of GA in sGTND. The changes in intracellular isocitrate and fumarate levels were consistent with those of malate (Fig. 6H and I). These results demonstrate that the combination of sGTND and UTMD can effectively reduce the levels of TCA cycle metabolites, thereby inhibiting the TCA cycle.

Collectively, the combination of sGTND and UTMD effectively inhibited the TCA cycle through a coordinated mechanism involving NRF2 activation and G6PD suppression. This dual approach resulted in upregulated expression of xCT/SLC7A11, ME1 and IDH1 proteins, decreased intracellular NADPH with concomitant elevation of the NADP+/NADPH ratio, and depletion of key TCA cycle substrates (malate, isocitrate, and fumarate), ultimately achieving TCA cycle suppression.

### *In vitro* antitumor efficacy

**Fig. 7:**
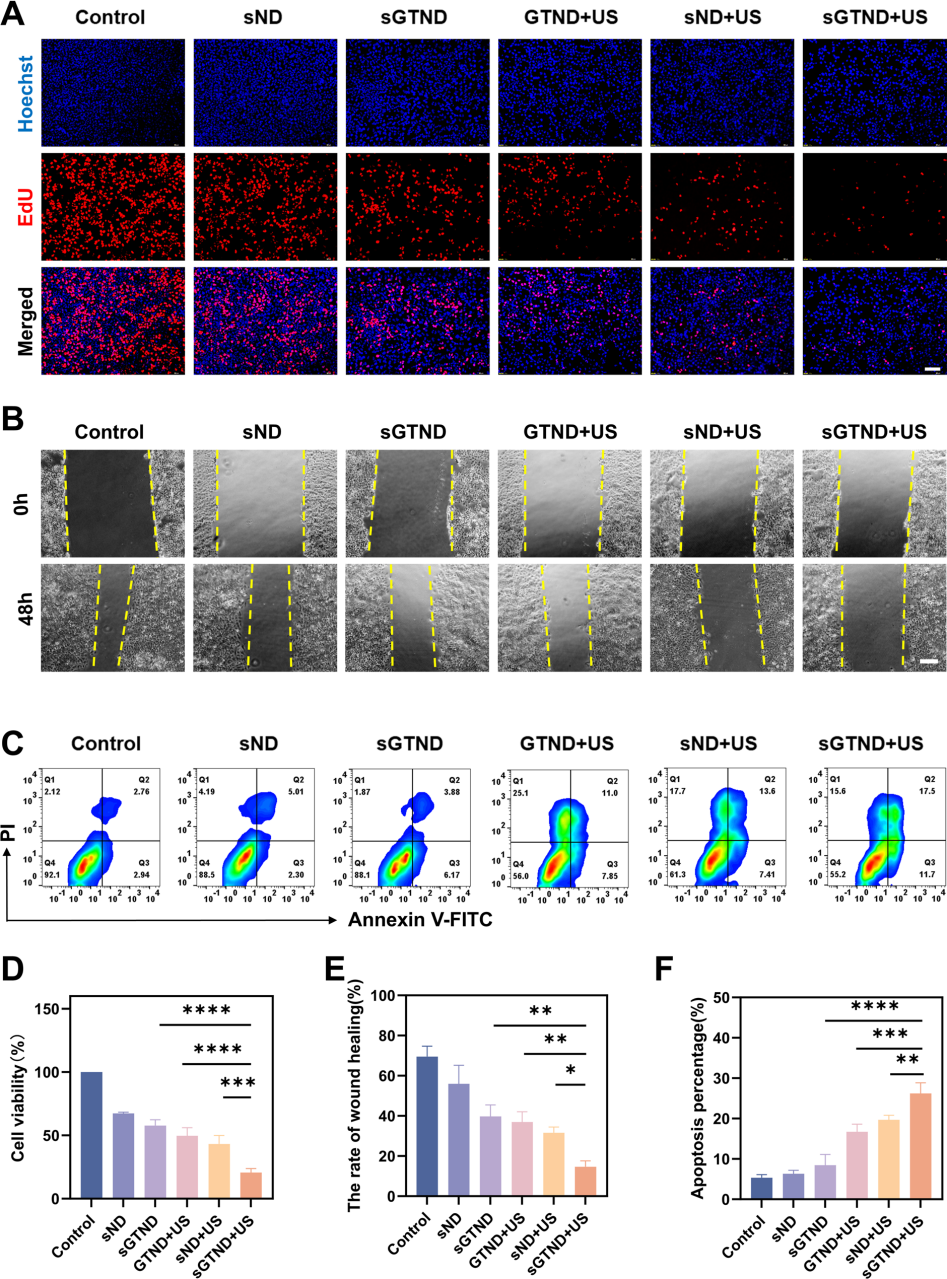
Antitumor efficacy of sGTND combined with UTMD in vitro. (**A**) Fluorescence images of EdU-positive Huh-7 cells under different treatments. Scale bar: 100 μm. (**B**) Images of Huh-7 cell migration under different treatments for 48 h. Scale bar: 100 μm. (**C**) Annexin V-FITC/PI double-staining analysis under different treatments. (**D**) Cell viability under different treatments measured by the CCK-8 assay. (**E**) Quantitative analysis of cell migration from B. (**F**) Quantitative analysis of cell apoptosis from C. All results are expressed as mean ± SD (*n* = 3). *p < 0.05, **p < 0.01, ***p < 0.001 and ****p < 0.0001

To investigate the cytotoxic effects of sGTND in combination with UTMD on HCC cells, the viability of cells in different treatment groups was assessed using the CCK8 assay. As depicted in Fig. 7D, the sGTND + US group exhibited the lowest cell viability rate, at 20.64 ± 3.16%. In contrast, the sND + US group, which lacks targeting ability and cannot release GA under UTMD, had a higher cell viability rate of 43.31 ± 6.57%. The cell viability rates for the GTND + US, sGTND, and sND groups were 49.64 ± 6.45%, 57.84 ± 4.49%, and 67.38 ± 0.10%, respectively. The lower cell viability rate in the GTND + US group compared to the sGTND and sND groups may be attributed to the partial inhibition of tumor cell TCA by the activation of the NRF2 pathway alone. Consistent with the CCK8 results, the cell proliferation assay revealed that the sGTND + US group had the lowest percentage of positive cells (Fig. 7A), indicating that the combined treatment effectively suppressed the proliferation of HCC cells.

The scratch assay was employed to evaluate the impact of sGTND in combination with UTMD on the migration of HCC cells in vitro. As shown in Figs. 7B and E, after 48 h, the cell migration rates for the control, sND, sGTND, GTND + US, sND + US, and sGTND + US groups were 69.51 ± 5.19%, 55.99 ± 9.20%, 39.70 ± 5.79%, 36.94 ± 5.07%, 31.62 ± 2.87%, and 14.74 ± 2.93%, respectively. The sGTND + US group had a significantly lower cell migration rate (*p* < 0.05). To assess the effect of the combined treatment on cell apoptosis, cells were subjected to different treatments and then stained with Annexin V-FITC and PI. As illustrated in Figs. 7C and F, the sGTND + US group had the highest total percentage of apoptotic cells, at 26.25 ± 2.620%, indicating that the combination of sGTND and UTMD significantly promoted cell apoptosis. Collectively, these findings demonstrate that the combination of sGTND and UTMD significantly inhibits the viability, proliferation, and migration of HCC cells while promoting apoptosis.

### *In vivo* antitumor efficacy

**Fig. 8:**
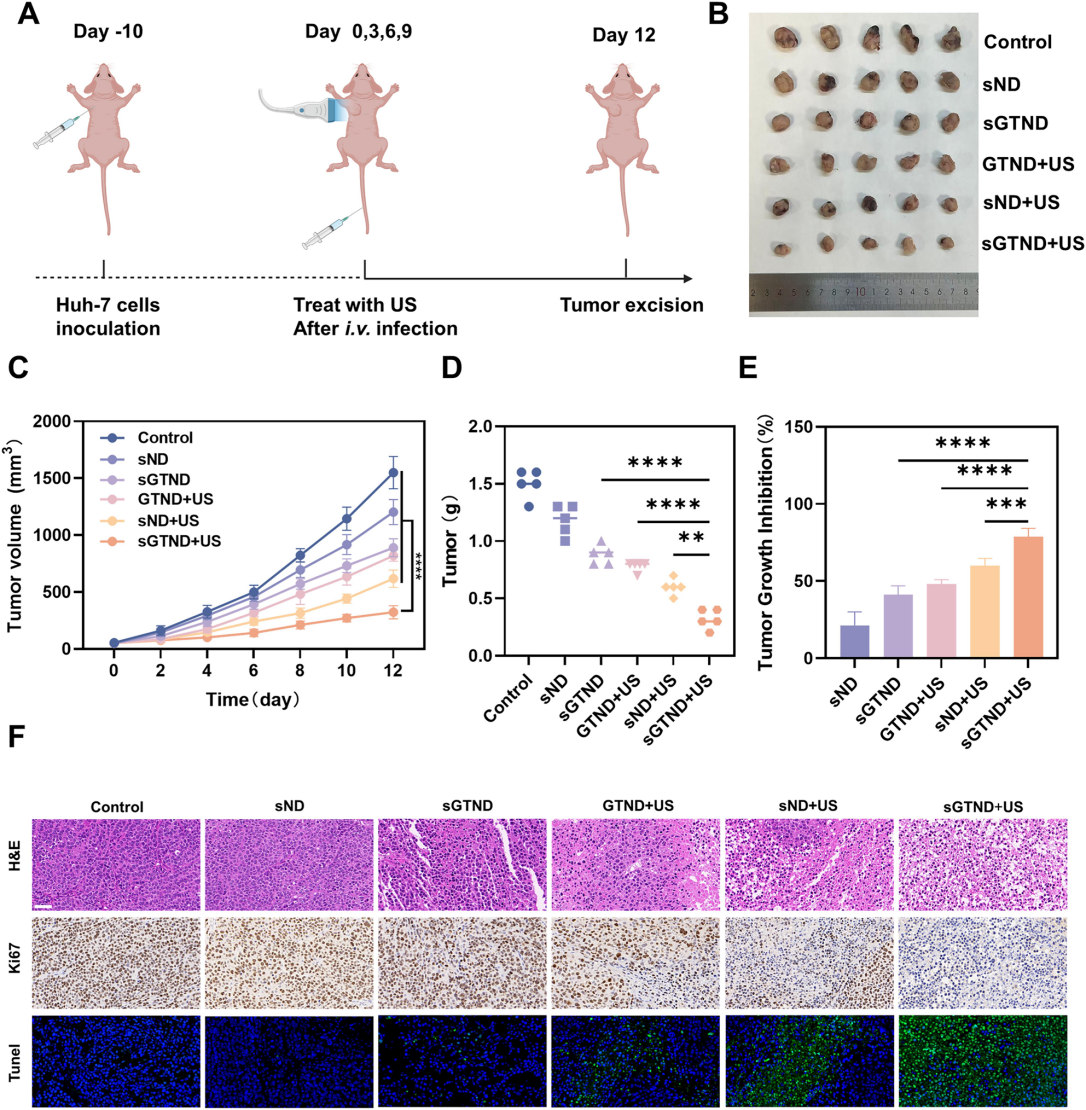
Suppression of Huh-7 tumor growth by sGTND combined with UTMD in vivo. (**A**) Schematic illustration of the Huh-7 tumor model and treatment regimen. (**B**) Representative tumor images from Huh-7 tumor-bearing mice in each treatment group after therapy. (**C**) Tumor volume curves for different treatment of mice within 12 days. (**D**) The weight of tumors excised from each group of mice at the end of the experiment. (**E**) Tumor inhibition rate in each group following treatment. (**F**) H&E staining, Ki67 immunostaining, and TUNEL assay images of tumor tissues from each group. Scale bar: 50 μm. All data are presented as mean ± SD (*n* = 5). *p < 0.05 and ****p < 0.0001

To evaluate the in vivo antitumor efficacy of sGTND combined with UTMD, an immunodeficient mouse model of HCC xenograft was established and treated in different groups (Fig. 8A). After the treatment was completed, the mice were sacrificed, and the tumor tissues were removed, photographed, and weighed (Fig. 8B). During the treatment, the tumor volume increased in all groups. However, compared with the control group, the tumor growth rate was slower in all treatment groups, with the slowest growth rate observed in the sGTND + US group (Fig. 8C), which also had the smallest tumor weight at the end of the treatment (Fig. 8D). The tumor growth rate inhibition rate of the sGTND group, GTND + US group, sND + US group, and sGTND + US group were 21.33%,41.33%,48%,60%,78.67% (Fig. 8E). These results indicate that the combination of sGTND and UTMD effectively inhibits tumor growth in vivo.

H&E, Ki67, and TUNEL staining further confirmed the inhibitory effect of sGTND combined with UTMD on tumor growth. As shown in Fig. 8F, in H&E staining, the tumor cells in the control group grew densely with large and deeply stained nuclei. In contrast, varying degrees of damage were observed in the tumor tissues of the other groups, with the sGTND + US group showing significant nuclear pyknosis, karyorrhexis, and karyolysis, indicating the most severe damage. Ki67 and TUNEL staining revealed that cell proliferation was significantly reduced and apoptosis was markedly increased in the sGTND + US group. These results demonstrate that the combined therapy effectively suppresses tumor cell proliferation and induces apoptosis in vivo, leading to significant tumor growth inhibition. What is more, immunohistochemical analysis of tumor specimens revealed that the expression patterns of NRF2, xCT/SLC7A11, and the TCA cycle-related markers ME1 and IDH1 were generally consistent with those observed in vitro (Figure S6), further validating the molecular mechanism by which sGTND combined with UTMD suppresses TCA activity.

## Conclusion

In this study, leveraging the metabolic characteristics of TCA cycle dependency in HCC, we designed a targeted ND with dual responsiveness to US and ROS. First, we designed and synthesized a novel GTC polymer by connecting GA and CMC via a ROS-responsive TK bond, as confirmed by FT-IR and ^1^H-NMR analyses. Then, using this GTC polymer as the shell and PFH as the core, we loaded PEI-siG6PD to construct sGTND, with an average particle size of 301.7 ± 22.11 nm. These NDs responded to ROS to enable controlled drug release and to US for CEUI capabilities, and they also exhibited excellent uniformity, biocompatibility and tumor-targeting properties. Finally, in vitro and in vivo results demonstrated that the combination of sGTND and UTMD effectively suppressed the TCA cycle through the synergistic role of NRF2 activation and G6PD inhibition, thereby inhibiting HCC growth. Overall, the sGTND+UTMD strategy represents a promising therapeutic approach for HCC.

## Supplementary Information

Supplementary Material 1.

## Data Availability

The data that support the findings of this study are available from the corresponding author upon reasonable request.
