# Supplementary material for: Ultrasound and ROS-responsive nanodroplets inhibit TCA cycle in hepatocellular carcinoma
Source: J Nanobiotechnology. 2026 Mar 1;24:317. doi: 10.1186/s12951-026-04190-y (PMC13059381; doi:10.1186/s12951-026-04190-y)
Supplement: Supplementary file 1 — Supplementary Material 1. [file 12951_2026_4190_MOESM1_ESM.docx]

**Supporting Information**

**Ultrasound and ROS-Responsive Nanodroplets inhibit TCA Cycle in Hepatocellular Carcinoma**

Ting Zhao^a, 1^, Lu Guo^a, 1^, Ning Cong^a,1^, Yading Zhao^a^, Xiaoxuan Wang^a^, Xinyu Zeng^a^, Suyun Li^a^, Rui Liu^a^, Shuting Huang^a^, Yuye Fu^a^, Jie Li^a,b*^

^*^Correspondence: Jie Li, [jieli@email.sdu.edu.cn](mailto:jieli@email.sdu.edu.cn)

^a^Department of Ultrasound, Qilu Hospital of Shandong University, Jinan, Shandong 250012, China

^b^Department of Ultrasound, Qilu Hospital (Qingdao) of Shandong University, Qingdao, Shandong 266035, China

^1^Ting Zhao, Lu Guo and Ning Cong contributed equally to this work.

This part included: Figure S1-S9


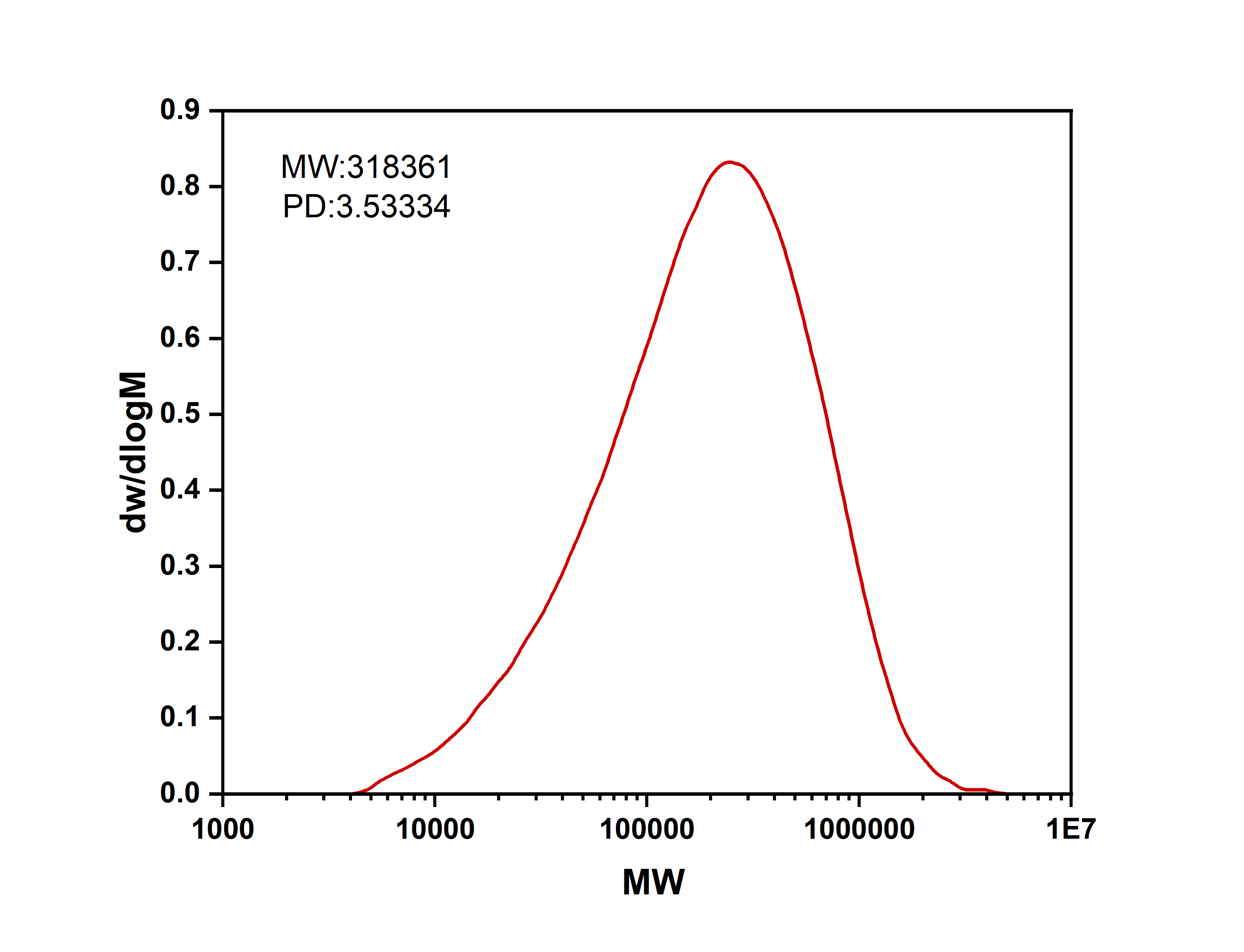


**Figure S1**. GPC spectrum of CMC.

Figure S2. Long-term stability assay for sGTND. (A) Optical microscopy images of sGTND on day 0 and after 7 days of storage. (B) Particle size of sGTND after 7 days of storage. (C) Comparison of particle size between day 0 and day 7. Data are presented as mean ± SD (n=3). ns: not significant.


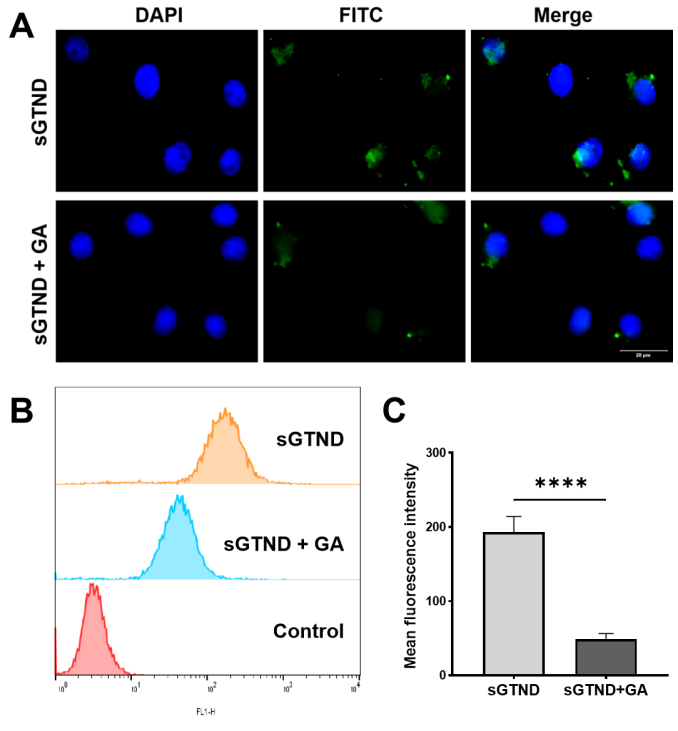


Figure S3. GA blocking assay for sGTND. (A) Fluorescence microscopy images and (B) FCM analysis showing the cellular uptake of sGTND by Huh-7 cells after GA pretreatment for 1 h. (C) Quantification of the FCM results shown in (B). Data are presented as mean ± SD (n=3). *****p* < 0.0001.


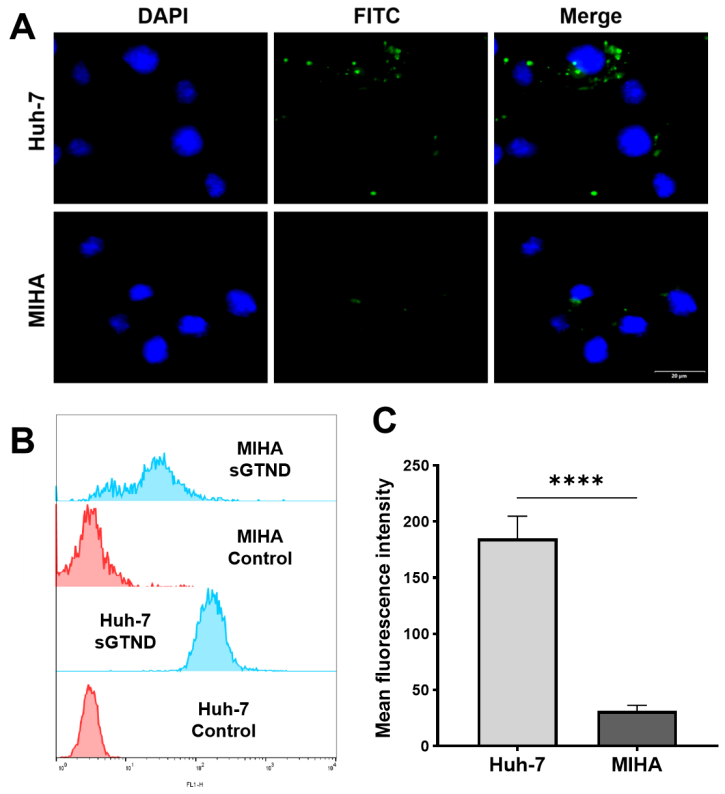


Figure S4. *In vitro* cellular uptake of sGTND by normal hepatocytes (MIHA) and HCC cells (Huh-7). (A) Fluorescence microscopy images and (B) Flow cytometry analysis showing the cellular uptake of sGTND by Huh-7 and MIHA cells. (C) Quantification of the mean fluorescence intensity from B. Data are presented as mean ± SD (n=3). *****p* < 0.0001.


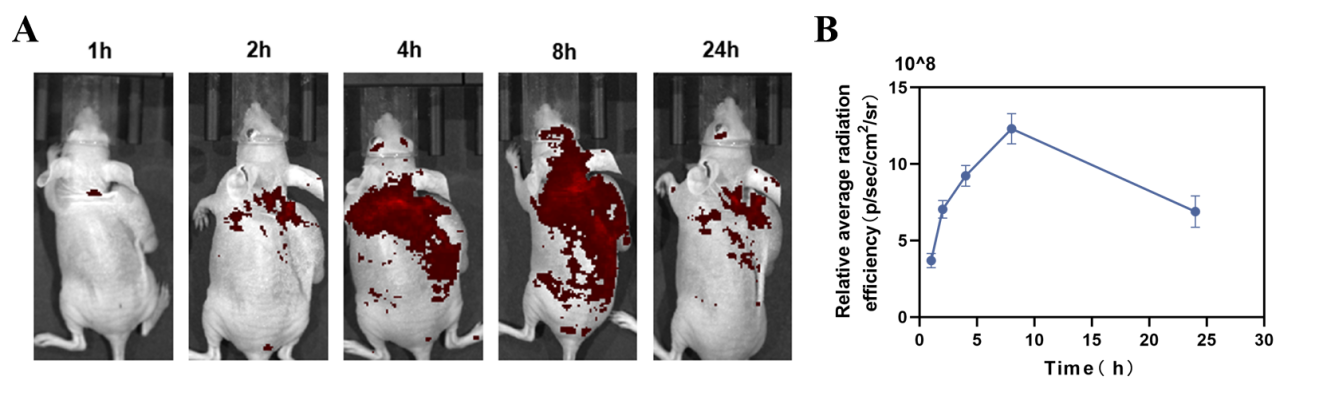


Figure S5. *In vivo* biodistribution of sGTND in Huh-7 tumor-bearing mice. (A) Representative fluorescence images at 1, 2, 4, 8, and 24 h after intravenous injection of sGTND. (B) Quantitative analysis corresponding to the fluorescence images shown in (A).


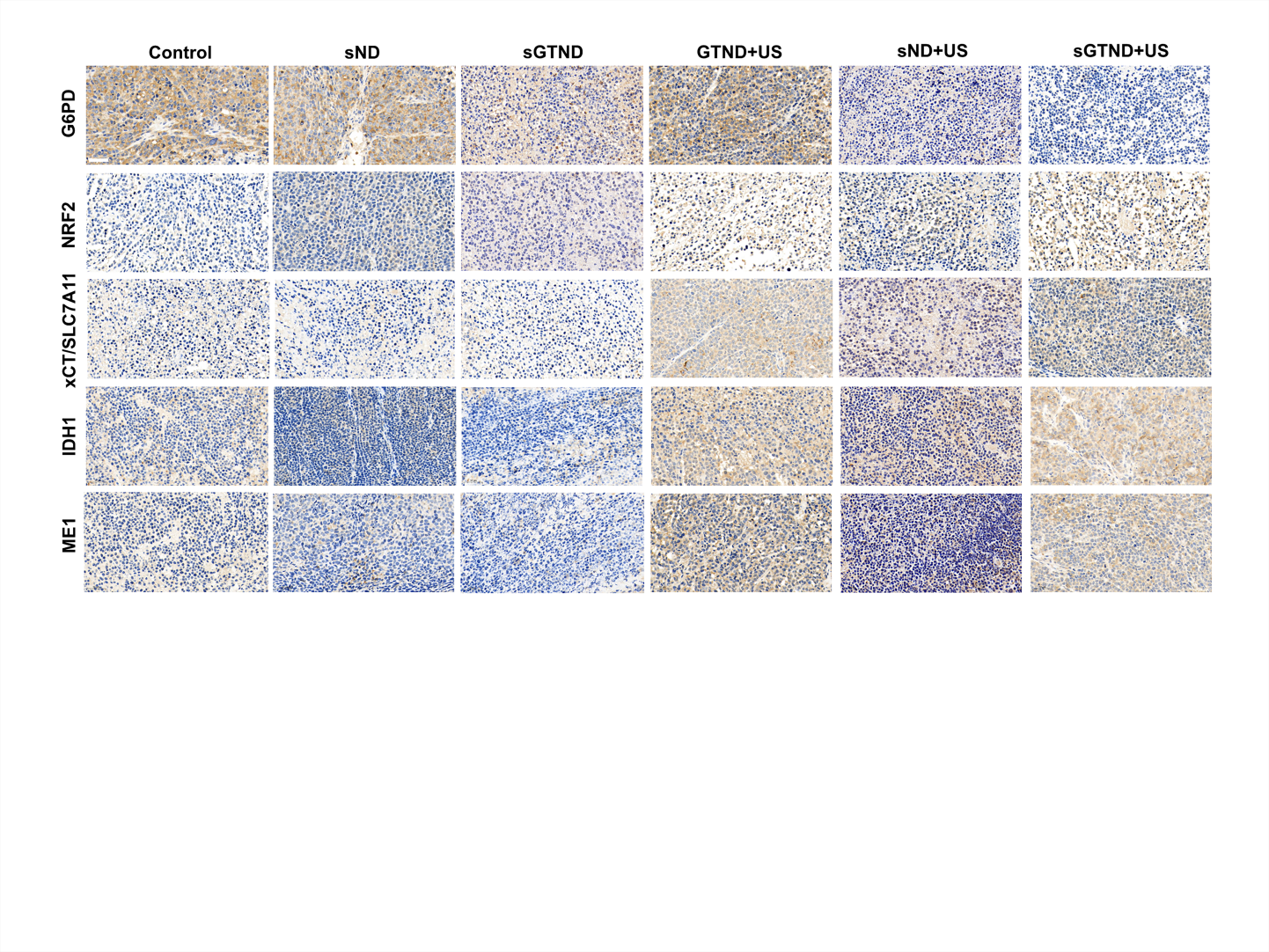


Figure S6. Immunohistochemical analysis of G6PD, NRF2, xCT/SLC7A11, IDH1, and ME1 expression in mouse tumor tissues. Scale bar: 50 μm.


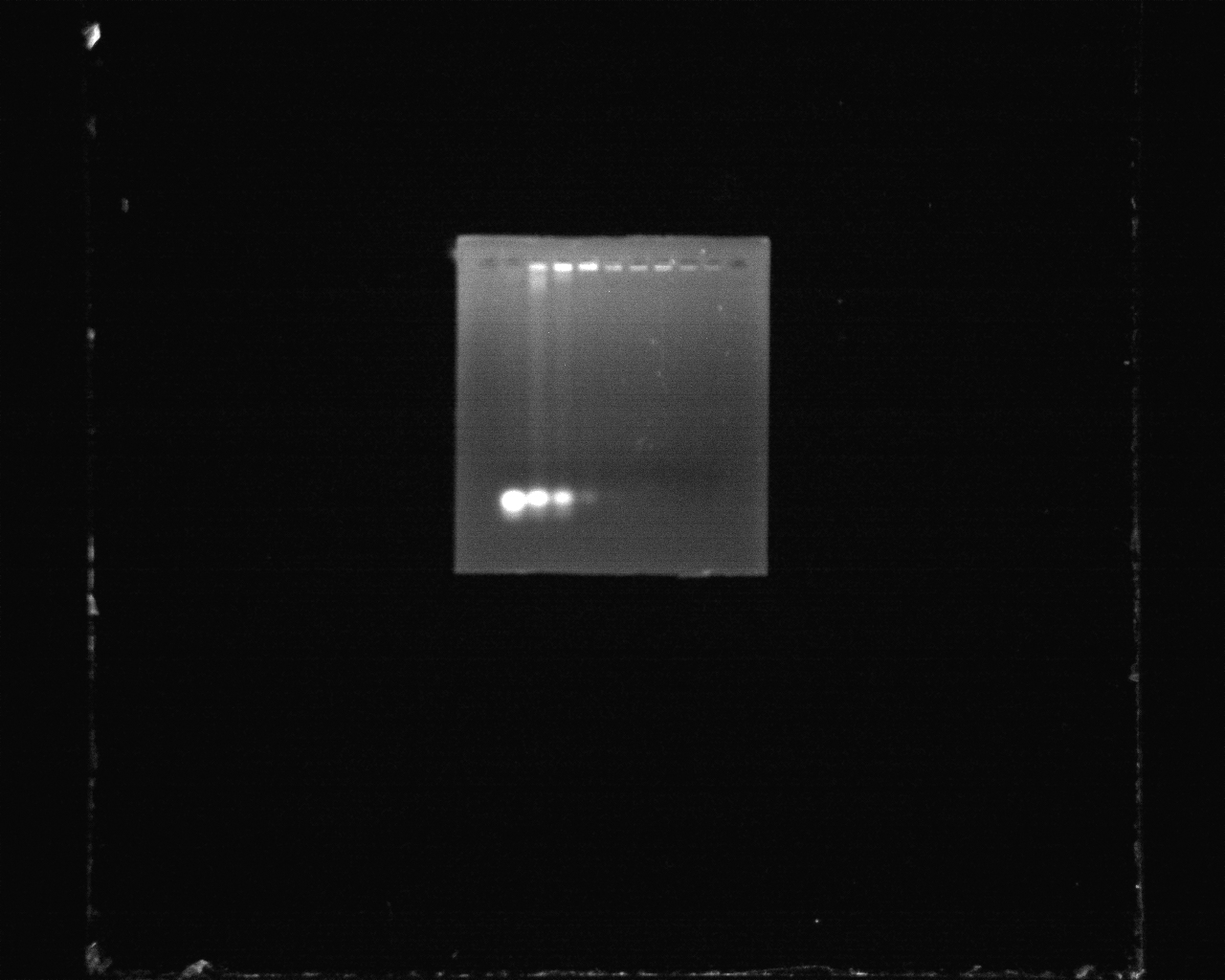


Figure S7. Uncropped agarose gel electrophoresis image corresponding to Figure 2E.


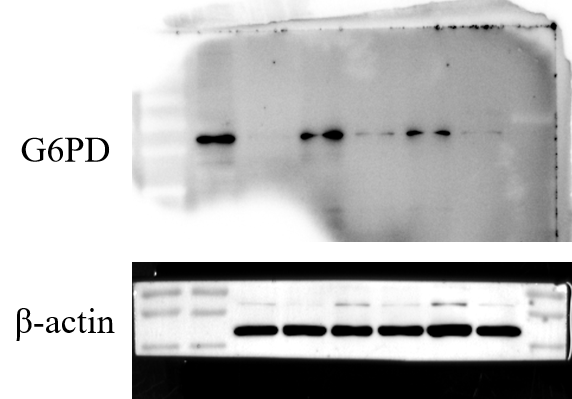


Figure S8. Western blot raw images corresponding to Figure 5A, including G6PD and β‑actin.


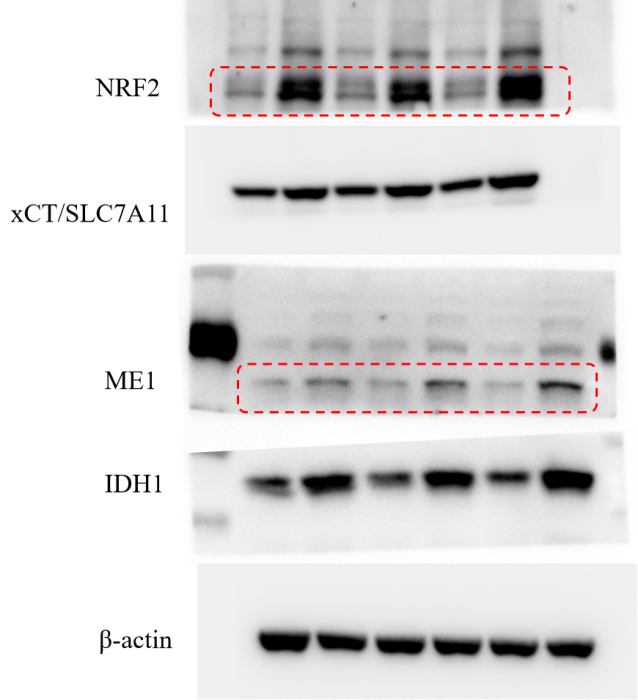


Figure S9. Western blot raw images corresponding to Figure 6A, including NRF2, xCT/SLC7A11, ME1, IDH1, and β‑actin. The red rectangles indicate the bands corresponding to the target proteins.
